# Influence of Hard Segment Content and Diisocyanate Structure on the Transparency and Mechanical Properties of Poly(dimethylsiloxane)-Based Urea Elastomers for Biomedical Applications

**DOI:** 10.3390/polym13020212

**Published:** 2021-01-09

**Authors:** Natascha Riehle, Kiriaki Athanasopulu, Larysa Kutuzova, Tobias Götz, Andreas Kandelbauer, Günter E. M. Tovar, Günter Lorenz

**Affiliations:** 1 Reutlingen Research Institute, Reutlingen University, Alteburgstr. 150, 72762 Reutlingen, Germany; natascha.riehle@gmx.de (N.R.); kiriaki.athanasopulu@reutlingen-university.de (K.A.); larysa.kutuzova@reutlingen-university.de (L.K.); 2 Center for Process Analysis & Technology (PA&T), School of Applied Chemistry, Reutlingen University, Alteburgstrasse 150, 72762 Reutlingen, Germany; andreas.kandelbauer@reutlingen-university.de; 3 Institute of Interfacial Process Engineering and Plasma Technology IGVP, University of Stuttgart, Nobelstr. 12, 70569 Stuttgart, Germany; tobias.goetz@igvp.uni-stuttgart.de (T.G.); guenter.tovar@igvp.uni-stuttgart.de (G.E.M.T.); 4 Fraunhofer-Institute for Interfacial Engineering and Biotechnology IGB, Nobelstr. 12, 70569 Stuttgart, Germany

**Keywords:** segmented polyurea elastomers, poly(dimethylsiloxane), structure-property relationship, diisocyanate structure, transparency, mechanical properties, tensile hysteresis, in vitro cytotoxicity, biomedical applications, biomaterials

## Abstract

The effect of hard segment content and diisocyanate structure on the transparency and mechanical properties of soft poly(dimethylsiloxane) (PDMS)-based urea elastomers (PSUs) was investigated. A series of PSU elastomers were synthesized from an aminopropyl-terminated PDMS (${\bar{M}}_{n}$: 16,300 g·mol^−1^), which was prepared by ring chain equilibration of the monomers octamethylcyclotetrasiloxane (D_4_) and 1,3-bis(3-aminopropyl)-tetramethyldisiloxane (APTMDS). The hard segments (HSs) comprised diisocyanates of different symmetry, i.e., 4,4′-methylenebis(cyclohexyl isocyanate) (H_12_MDI), 4,4′-methylenebis(phenyl isocyanate) (MDI), isophorone diisocyanate (IPDI), and *trans*-1,4-cyclohexane diisocyanate (CHDI). The HS contents of the PSU elastomers based on H_12_MDI and IPDI were systematically varied between 5% and 20% by increasing the ratio of the diisocyanate and the chain extender APTMDS. PSU copolymers of very low urea HS contents (1.0–1.6%) were prepared without the chain extender. All PSU elastomers and copolymers exhibited good elastomeric properties and displayed elongation at break values between 600% and 1100%. The PSUs with HS contents below 10% were transparent and became increasingly translucent at HS contents of 15% and higher. The Young’s modulus (YM) and ultimate tensile strength values of the elastomers increased linearly with increasing HS content. The YM values differed significantly among the PSU copolymers depending on the symmetry of the diisocyanate. The softest elastomer was that based on the asymmetric IPDI. The elastomers synthesized from H_12_MDI and MDI both exhibited an intermediate YM, while the stiffest elastomer, i.e., that comprising the symmetric CHDI, had a YM three-times higher than that prepared with IPDI. The PSUs were subjected to load–unload cycles at 100% and 300% strain to study the influence of HS morphology on 10-cycle hysteresis behavior. At 100% strain, the first-cycle hysteresis values of the IPDI- and H_12_MDI-based elastomers first decreased to a minimum of approximately 9–10% at an HS content of 10% and increased again to 22–28% at an HS content of 20%. A similar, though less pronounced, trend was observed at 300% strain. First-cycle hysteresis among the PSU copolymers at 100% strain was lowest in the case of CHDI and highest in the IPDI-based elastomer. However, this effect was reversed at 300% strain, with CHDI displaying the highest hysteresis in the first cycle. In vitro cytotoxicity tests performed using HaCaT cells did not show any adverse effects, revealing their potential suitability for biomedical applications.

## 1. Introduction

Segmented polyurethanes (PUs) and polyureas (PUrs) are a broad class of polymers whose mechanical properties range from those of very soft elastomers to those of rigid thermoset-like materials with high mechanical strengths. This versatility is a result of the segmented structure of these polymers, which consist of alternating blocks of hard and soft segments. The soft segment determines the elastomeric properties and typically comprises macrodiols or diamines based on polyesters, polyethers, aliphatic polycarbonates, or, more recently, polybutadienes [1], polyisobutylenes [2], or poly(dimethylsiloxane)s with molecular weights between ca. 900 and 4000 g·mol^−1^ [3]. The hard segment (HS) consists of a diisocyanate and a low-molecular-weight diol or diamine chain extender and governs both the thermoplastic properties and the mechanical strength of the final polymer. Due to the inherent thermodynamic incompatibility of these segments, PUs and, in particular, PUrs display morphologies in which the HSs aggregate into hard domains that are phase separated from a surrounding matrix of soft domains [3].

The mechanical properties of PUs can be tailored according to required specifications by careful consideration of the chemistries, structures, and compositions of the macrodiol, diisocyanate, and chain extender moieties [4]. Therefore, PUs are widely applied in the fields of medical and biomedical engineering [5] for blood bags, tubing and valves, cardiac assist devices [6], drug-releasing coatings [7,8], antibacterial [9] and anti-biofilm coatings for urological catheters [10], and wound dressings [11].

The chemical stability of the soft segment is an important issue when considering the use of PUs in long-term implants like breast implant coatings [12] or artificial heart valves [13]. Polyester-based PUs degrade quite rapid in vivo as a result of hydrolytic cleavage of the ester bonds. For this reason, they are often utilized for short-term and biodegradable applications, e.g., scaffolds for meniscal reconstruction [14] or for the growth of human retinal epithelium cells after age-related macular degeneration (AMD) [15]. Conversely, poly(ether urethane)s, particularly PUs based on poly(tetramethylene oxide) (PTMO), are prone to oxidative degradation by environmental stress cracking and transition-metal-ion oxidation [16]. Therefore, poly(dimethylsiloxanes) (PDMSs), which are well known for their excellent biocompatibilities and chemical stabilities, are incorporated as co-macrodiols in polyether- and poly(carbonate urethane)s in order to enhance their biostabilities [17,18]. For instance, Gunatillake’s group conducted systematic research on designing biostable PUs. They employed a mixed soft segment based on bishydroxyethoxypropyl PDMS along with the polyether poly(hexamethylene oxide) (PHMO) and successfully improved the biostability of the synthesized PU relative to a soft medical grade poly(ether urethane) [19]. Further research and development of these PUs led to their commercialization and approval as biomaterials for gastroenterological implants, lead insulators, and tri-leaflet heart valves [20].

An important factor for the design of mechanically stable PUs and PUrs is the composition and structure of the HS. PDMS-based PUs typically exhibit a high degree of microphase separation due to the strong hydrophobic nature of this macrodiol and the high polarity of the urethane groups [21]. However, most PDMS-based PUs lack sufficient mechanical stability, particularly when a high-molecular-weight PDMS is applied. However, Yilgör et al. showed that the synthesis of PDMS-based PUrs having reasonable mechanical properties, even at low HS ratios, is possible [22]. This difference in mechanical strength results from the strong bidentate intermolecular hydrogen bonding of the urea groups. Unlike real elastomers, which gain their mechanical strength from the chemical cross-linking of the polymer chains, the mechanical properties of PUs are largely dependent on the strength and effectiveness of their physical cross-links. Hence, the effects of the diisocyanate structure and symmetry on the morphology and tensile properties of PUs, polyurethaneureas, and PUrs has been addressed in various studies. For example, Adhikari et al. and Saralegi et al. both studied the influence of the symmetric *trans*-*trans*-4,4′-methylenebis(cyclohexyl isocyanate) (H_12_MDI) isomer ratio on the mechanical properties and microphase separation of PUs [23,24]. They found that microphase separation in the PUs increases due to a higher HS ordering. This led to an increase in Young’s modulus (YM), hardness, and thermal stability. However, tensile strength seemed to be dependent on both the degree of HS crystallinity and the ability of the soft segment to form ordered structures upon strain [24]. Similarly, Joseph et al. studied the effect of varying the *cis/trans*-isomer distribution in 1,4-cyclohexane diisocyanate (CHDI)-based poly(ether urethanes) and also determined an increase in hardness and HS melting temperature when the ratio of *trans*-CHDI was increased [25]. HS crystallinity plays an important role in the mechanical properties of PUs, particularly on their tensile hysteresis behavior. This effect was thoroughly studied by Prisacariu et al. on a set of PUs based on 4,4′-methylenebis(phenyl isocyanate) (MDI), 4,4′-dibenzyl diisocyanate (DBDI), and equimolar mixtures of these diisocyanates [26]. In contrast to MDI, in which the phenyl rings are linked by a methylene bridge, DBDI can adopt a more symmetric conformation due to rotation around the ethylene bridge, which promotes the formation of crystalline hard domains [27]. The stress-induced disruption of such domains was found to be accompanied by a higher hysteresis compared to that for HSs formed by MDI or diisocyanate mixtures. Morphological studies on non-chain-extended poly(ether urethane)s and PUrs containing diisocyanates of different symmetry in their HSs have been conducted, particularly by Yilgör and co-workers. They applied atomic force microscopy, small-angle X-ray scattering (SAXS), wide-angle X-ray scattering (WAXS), dynamic mechanical analysis, and tensile measurements to investigate the influence of hydrogen bonding and diisocyanate symmetry on microphase separation and morphology in these elastomers [28,29]. While the PUs, which contained the asymmetric diisocyanates H_12_MDI, MDI, toluene diisocyanate (TDI), and *m*-phenylene diisocyanate (*m*-PDI), were tacky, the analogous urea copolymers all displayed a microphase separated morphology and behaved as elastomeric materials. Furthermore, WAXS studies on urea copolymers revealed crystalline HSs except in those that contained isomeric mixtures (H_12_MDI, TDI). As a result, the stress in urea copolymers was higher compared to that in the urethanes. However, the first-cycle hysteresis values (300% strain) of the copolymers were generally very high (between 72% and 89%). Interactions between HSs were also studied utilizing quantum mechanical calculations, dissipative particle dynamics (DPDs), and density functional theory (DFT) simulations [30,31]. The structures of model HSs as well as calculated hydrogen bond energies and distances revealed the presence of long-range-ordered hydrogen bonds in PUs and PUrs comprising symmetric diisocyanates (CHDI, HDI, *p*-PDI). This organization of hydrogen bonds allows tight packing of the chains and promotes the formation of crystalline domains, which corresponds to the reported results, such as high YM, tensile strength, and high hysteresis [30,31].

Most of these structure morphology relationship studies were performed on polyether-based PUs and PUrs that were synthesized either without chain extenders or with symmetric chain extenders such as ethylene or butylene glycol. However, the etheric oxygen can interact with urethane/urea groups by hydrogen bonding, thus influencing morphology and mechanical properties [31]. Furthermore, the HS content and the soft segment molecular weight in the elastomers also have effects on mechanical properties [22].

Elucidation of the hard domain morphology was the main focus of the studies cited above. However, the resulting effect on tensile hysteresis behavior was often largely ignored in those investigations. Moreover, the optical appearance of the synthesized PUs was seldom addressed in this structure property context. Therefore, a study into both the influence of the diisocyanate structure and the effect of increasing the HS content on the mechanical properties and the transparency of PDMS-based urea elastomers is called for.

**Figure 1:**
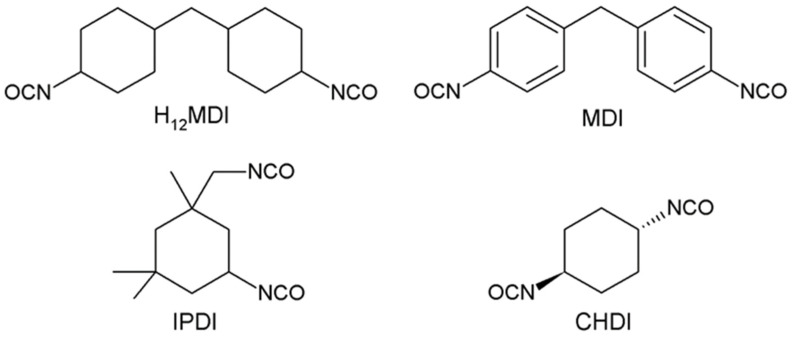
Diisocyanates used in the synthesis of PDMS-based urea elastomers in this study: 4,4′-methylenebis(cyclohexyl isocyanate) (H_12_MDI), 4,4′-methylenebis(phenyl isocyanate) (MDI), isophorone diisocyanate (IPDI), and *trans*-1,4-cyclohexane diisocyanate (CHDI).

Our previous study demonstrated that very soft aliphatic polysiloxane-based urea elastomers are obtained when the molecular weight of the PDMS soft segment is increased. These elastomers were intended for application as intraocular lenses and exhibit a transmission of approximately 90% between 750 and 500 nm until a PDMS molecular weight of 18,000 g·mol^−1^ [32]. The current work represents a significant extension of our earlier study published in Eur Poly. J [32]. In the present work, a number of different hard segment concentrations were studied, and, in contrast to the previous paper, several types of diisocyanates (see structures in Figure 1) were investigated as well (H_12_MDI, IPDI, CHDI, MDI). Hence, the current work describes a much wider range of materials that were studied as potentially suitable biomaterials specifically for intraocular lens applications. For intraocular lens applications the optical properties of the materials are of special importance, in addition to the mechanical properties; therefore, the transparency of the produced materials and their biocompatibility were studied in detail.

This study presents the synthesis and characterization of a series of PDMS-based PUr elastomers (PSUs), which were prepared from an aminopropyl-terminated PDMS with a molecular weight of approximately 16,300 g·mol^−1^ and the diisocyanates H_12_MDI, isophorone diisocyanate (IPDI), CHDI, and MDI. An aminopropyl-terminated disiloxane (APTMDS) was applied as a chain extender. To study the effect of the HS content on the transparency and mechanical properties of the PSU elastomers, the amount of HS was varied between 5% and 20% in aliphatic PSU elastomers prepared from H_12_MDI and IPDI. Furthermore, PSU copolymers were synthesized without the chain extender to investigate the influence of the diisocyanate structure on the mechanical properties of the PSU copolymers. The tensile hysteresis behavior of the PSU elastomers and copolymers was investigated in terms of their HS contents and the type of diisocyanate applied. For potential biomedical application of such PSU elastomers, e.g., in vascular prostheses, wound dressings, intraocular lenses, and soft coatings for medical devices, it is important that these materials do not show any adverse effects in contact with biological media and cells. Therefore, cell-medium extracts of the PSU elastomers were applied for in vitro cytotoxicity assessments to evaluate whether their low-molecular-weight leachables diminish cell viability.

## 2. Materials and Methods

### 2.1. Chemicals

APTMDS (97%) was purchased from ABCR GmbH (Karlsruhe, Germany). Tetrahydrofuran (THF) (anhydrous 99.8%, stabilized with butylated hydroxytoluene) was purchased from Alfa Aesar (Thermo Fisher GmbH, Karlsruhe, Germany). MDI (98%) was purchased from Sigma Aldrich Chemie GmbH (Taufkirchen, Germany). H_12_MDI and IPDI were kindly supplied by CSC Jäkle Chemie GmbH & Co. KG (Nürnberg, Germany). CHDI was synthesized in-house according to a method published elsewhere [33]. The purities of the applied diisocyanates (H_12_MDI, IPDI, and CHDI) were at least 99% as determined by the dibutylamine back titration method carried out according to DIN EN ISO 14896:2009-07. *α,ω*-Bis(3-aminopropyl)-PDMSs with molecular weights of ca. 16,300 g·mol^−1^ (see Table 1) were prepared by ring-chain equilibration of octamethylcyclotetrasiloxane (D_4_) and APTMDS according to a method described elsewhere [34].

PDMS and APTMDS were dried and degassed under vacuum before use. CHDI was purified by resublimation and distillation under vacuum before use. All other chemicals were used as received.

Human adult calcium high-temperature keratinocytes (HaCaT cells) were obtained from CLS Cell Lines Service GmbH (Eppelheim, Germany). Dulbecco’s modified Eagle’s medium (DMEM) (high glucose) and fetal bovine serum (FBS) were purchased from Thermo Fisher Scientific Life Technologies GmbH (Darmstadt, Germany). The tetrazolium dye [3-(4,5-dimethylthiazol-2-yl)-5-(3-carboxymethoxyphenyl)-2-(4-sulfophenyl)-2H-tetrazolium salt] (MTS) was purchased as a CellTiter96^®^ Aqueous One Solution cell proliferation assay from Promega GmbH (Mannheim, Germany).

### 2.2. Determination of Molecular Weight

#### 2.2.1. Titration

Number average molecular weights (${\bar{M}}_{n}$) of the prepared PDMSs (see Table 1) were determined by titration of the amino end groups in THF with 0.1 N HCl using bromophenol blue (a detailed procedure is given in [35]). The molecular weight was calculated from an average of four titrations and was used for calculating the reaction stoichiometry of the subsequent PSU syntheses.

#### 2.2.2. Size-Exclusion Chromatography

Size-exclusion chromatography (SEC) studies were performed on a 1260 Infinity II GPC-SEC analysis system (Agilent Technologies Deutschland GmbH, Waldbronn, Germany) equipped with 2 PSS SDV columns (Polymer Standards Service) and a refractive index detector. PSU elastomers were dissolved in THF and filtered solutions were measured at 40 °C and a flow rate of 0.5 mL/min. Polystyrene standards were used for calibration.

#### 2.2.3. Determination of Intrinsic Viscosity

Intrinsic viscosity ([*η*]) values of the PSU elastomers were determined from PSU solutions in THF using an Ubbelohde viscometer. For each PSU elastomer, measurements were performed on four solutions with different concentrations (c) in the range 0.5–2.0 g/dL at 25 °C. Solutions were filtered through a PTFE-membrane filter prior to measurement to exclude any particles. Values for reduced viscosity (*η*_red_. = *η*_spec_./c) were calculated as averages from 10 replicate measurements. Standard deviations of the flow times were ≤0.7%. Values for [*η*] were obtained as the intercepts in plots of reduced viscosity (*η*_red._) versus concentration. (See Huggins plots in Figure A2 in Appendix B).

### 2.3. Characterization of Molecular Weight

PSU elastomers were prepared from the diisocyanates H_12_MDI, MDI, IPDI, and CHDI according to the prepolymer method. Syntheses were carried out in THF at room temperature or, in the cases of MDI and CHDI, at approximately 0 °C without a catalyst. The HS contents (see Equation (1)) of the PSU elastomers synthesized from H_12_MDI and IPDI were systematically varied between approximately 1.5% (PSU without chain extender) and 20% in steps of 5%. The labeling convention for the PSU elastomers is as follows: PSU diisocyanate-HS content (%), e.g., PSU H_12_MDI-1.6%.

(1) $$\mathrm{HS}\;\mathrm{content}\;\mathrm{in}\;\mathrm{PSU}\;\%\;=\;\frac{m\;(\mathrm{diisocyanate})\;(g)\;+\;m\;(\mathrm{APTMDS})\;(g)}{m\;(\mathrm{diisocyanate}\;+\;\mathrm{APTMDS}\;+\;\mathrm{PDMS})\;(g)}$$

A typical reaction procedure for the synthesis of a PSU copolymer (PSU H_12_MDI-1.6%) from a PDMS of ${\bar{M}}_{n}$: ca. 16,300 g·mol^−1^ was as follows: In a 250 mL four-neck, round-bottom reaction flask equipped with a PTFE centrifugal stirrer, dropping funnel, and nitrogen in- and outlets, 0.65 g (2.5 mmol) H_12_MDI was dissolved in 30 mL THF and added to the reaction flask. Then, 35.0 g (2.1 mmol) PDMS was dissolved in 100 mL THF and the PDMS solution was added dropwise to the H_12_MDI solution using the dropping funnel. Approximately 40 mL of THF, used to rinse the dropping funnel, was also added to the reaction mixture. The reaction progress was monitored using attenuated total reflection-Fourier transform infrared (ATR-FTIR) spectroscopy to measure the isocyanate absorption peak at 2265 cm^−1^. Portions of the PDMS (in steps of 1.5 g) dissolved in 10 mL THF were then added to the reaction mixture. After each addition, an IR spectrum was taken. Polyaddition was assumed complete when the isocyanate absorption peak had completely disappeared from the IR spectrum.

The synthesis of a PSU elastomer containing 20% HS (PSU H_12_MDI-20%) was performed as follows: First, 5.45 g (20.8 mmol) H_12_MDI, dissolved in 30 mL THF, was added to a round-bottom reaction flask. Then, 40.00 g (2.5 mmol) PDMS was dissolved in 100 mL THF and added dropwise to the H_12_MDI solution using a dropping funnel. Approximately 40 mL of THF, used to rinse the dropping funnel, was also added to the reaction mixture. The formation of the prepolymer was monitored via ATR-FTIR spectroscopy. Then, stoichiometric portions of APTMDS (total amount: 4.95 g; 18.3 mmol), each dissolved in 10 mL THF, were added dropwise to the prepolymer solution. The reaction progress was followed by monitoring the isocyanate absorption peak. The isocyanate peak disappeared from the IR spectrum after the last portion of APTMDS was added.

The PSU solutions were poured into PTFE-foil-covered glass petri dishes to evaporate the solvent under a fume-hood overnight. Finally, the PSU elastomers were further dried under vacuum at 80 °C for at least 24 h.

### 2.4. Preparation of PSU Elastomer Films for Measurement of Transparency and Mechanical Properties

Approximately 8 g of the PSU elastomer were cut into small pieces and dissolved in 200–300 mL CHCl_3_ with continuous stirring for at least 24 h using a magnetic stirrer bar. The homogenous polymer solution was then cast into a glass petri dish and covered with a perforated aluminum foil to slowly evaporate the solvent at room temperature in a well-ventilated location. Finally, the films were dried further in a vacuum chamber at 80 °C for approximately 12 h and carefully removed from the glass surface using a small thin spatula. The round edges were cut off and the films with final thicknesses between 0.33 and 0.40 mm were stored at approximately 23 °C within a transparent envelope until further characterization.

### 2.5. Measurement of Transparency

UV–Vis spectroscopy was applied to characterize the transparency of the synthesized PSU films. Film thicknesses ranged between 0.33 and 0.36 mm (see Table A8 in Appendix F). Measurements of the PSU films were performed on a Lambda1050 UV–Vis/NIR spectrophotometer (Perkin Elmer LAS GmbH, Rodgau, Germany) between 750 and 200 nm with a resolution of 2 nm and against air as the background. The spectra are displayed as the average from three measurements.

### 2.6. Measurement of Mechanical Properties

Tensile tests were performed on PSU films to determine YM, ultimate tensile strength (UTS), elongation at break, and mechanical hysteresis. Tensile and hysteresis measurements were performed on a Zwick model 81,565 tensile testing machine (Zwick GmbH & Co. KG, Ulm, Germany) using a 100 N load cell and testXpert II version 3.31 software. Dog-bone shaped specimens according to DIN 53504, type S2 [36] were die cut from these films and stored at 23 °C for at least 72 h until measurement. Prior to measurement, all samples were inspected under a cross-polarizer and confirmed free of internal stress. Samples with an original length (L_0_) of 20 mm were stretched until breaking with a crosshead speed of 25 mm/min after reaching a pre-load of 0.1 MPa.

The 10-cycle hysteresis behaviors of the PSU elastomers were investigated by stretching the samples to 100% or 300% elongation using a crosshead speed of 25 mm/min. After the samples had reached their desired elongation, they were immediately released at the same crosshead speed. The next cycle was started immediately after the crosshead had returned to the initial starting position.

Hysteresis for each cycle was calculated according to Equation (2):

(2) $$\mathrm{Hysteresis}\;\left(\%\right)\;=\frac{\mathrm{area}\;\mathrm{under}\;\mathrm{the}\;\mathrm{loading}\;\mathrm{curve}-\mathrm{area}\;\mathrm{under}\;\mathrm{the}\;\mathrm{recovery}\;\mathrm{curve}}{\mathrm{area}\;\mathrm{under}\;\mathrm{the}\;\mathrm{loading}\;\mathrm{curve}}\cdot 100\%$$

Measurements were performed at 23 ± 2 °C at a humidity of 55 ± 5% and results are reported as mean values of five measurements for tensile tests and three for each hysteresis measurement.

### 2.7. Measurement of In Vitro Cytotoxicity

The in vitro cytotoxicity of the PSU elastomers was measured according to the procedure described in DIN EN ISO 10993-5:2009 [37]. Cell-medium extracts of the elastomers were tested on HaCaT cells to evaluate whether their low-molecular-weight leachables have an adverse effect on cell proliferation. Cell viability was measured using an MTS cell proliferation assay after culturing the cells at 37 ± 1 °C and 5% CO_2_ with cell-medium extracts supplemented with 10% FBS.

HaCaT cells were cultured according to a protocol described elsewhere [36]. Cells were split at least four times before they were applied in the cytotoxicity assays.

Ethylene-oxide-sterilized samples of the PSUs and a biomedical-grade thermoplastic PU (Carbothane PC-3585A) reference material were weighed into falcon tubes and extracted with DMEM (without FBS) at an extraction ratio of 0.1 g/mL. Extraction was conducted at 37 °C and 5% CO_2_ atmosphere for 72 ± 2 h. Comparable volumes of fresh DMEM were pipetted into separate falcon tubes and subjected to the same extraction conditions. The DMEM without PSU was used as blank. After the extraction period, DMEM was immediately supplemented with 10% FBS. Fresh DMEM supplemented with 10% FBS was used as negative control. The positive control consisted of FBS-supplemented DMEM containing 1% sodium dodecyl sulfate.

HaCaT cells were seeded at a concentration of 20 × 10^3^ cells/well in 96-well microplates and incubated for 24 h at 37 °C and 5% CO_2_ atmosphere. Then, the cell medium was replaced with the extracts and controls, and the cells were incubated at 37 °C and 5% CO_2_ for a further 24 h. Just before the end of the incubation time, a stock solution of MTS was prepared by mixing the MTS dye and DMEM (without FBS) at 20 µL MTS + 100 µL DMEM for each well. After the incubation period, the extracts and controls were removed from the wells and 120 µL of the MTS stock solution was pipetted into each well. Additionally, the MTS solution was added to wells without cells for determination of the background.

The microplates were incubated for 4 h and measured with a microplate reader at a wavelength of 492 nm. In vitro cytotoxicity of each PSU elastomer was assessed by measuring three independent extracts with six replicates for each extract. Thus, 18 measurements in total were performed for each elastomer.

For analysis of cell proliferation, the background value of the MTS solution was subtracted from all absorbance values. All absorbance values of the positive control (no cell viability) were expressed as zero. The values of the negative control (serum-supplemented DMEM) were expressed as 100% cell proliferation. Absorbance values of the blank and the measured extracts were used to calculate percentage proliferation in comparison to the negative control (100% proliferation) and the positive control (0% proliferation). PSU extracts were defined as “not-cytotoxic” if the determined cell proliferation was over 81%.

## 3. Results and Discussion

### 3.1. Synthesis and Characterization of PDMS-Based PUr Elastomers

**Scheme 1:**
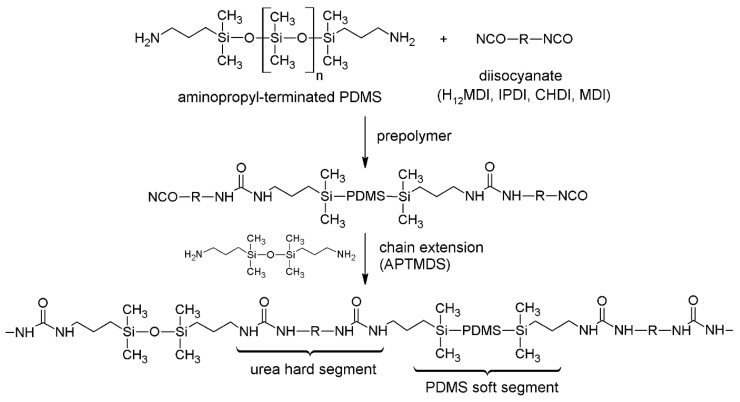
Synthesis of segmented PDMS-based polyurea elastomers via a two-step synthesis method. A prepolymer comprising active isocyanate groups is formed by reacting excess diisocyanate with amino-terminated PDMS. Segmented PSU elastomers, containing urea hard segments (HSs) and PDMS soft segments, are obtained after chain extension with APTMDS. For PSU copolymers synthesized without chain extender, segmented polymers consisting of alternating sequences of short HSs (exclusively diisocyanate) and long PDMS soft segments (>16,000 g·mol^−1^) are formed.

PSU elastomers were synthesized according to a two-step procedure (see Scheme 1). In the first step, the diisocyanate was reacted with the amino-terminated PDMS to yield a PSU prepolymer containing active isocyanate groups at each end of a PDMS chain. In the second step, the remaining isocyanate groups were reacted with the disiloxane-based chain extender APTMDS to build up a segmented polymer containing polar urea HSs and nonpolar PDMS soft segments.

In our previous report, the effect of PDMS molecular weight on the transparency and mechanical properties of urea elastomers was investigated [33]. The results revealed that very soft and transparent urea elastomers were obtained when a PDMS with a molecular weight of approximately 16,000 g·mol^−1^ was used. Therefore, the PSU elastomers in this study were prepared from PDMSs with molecular weights ranging between 16,300 and 16,500 g·mol^−1^. The HS content and the type of diisocyanate were altered in these PSU elastomers in order to systematically study their effects on the final transparencies and mechanical properties of the elastomers.

**Figure A1:**
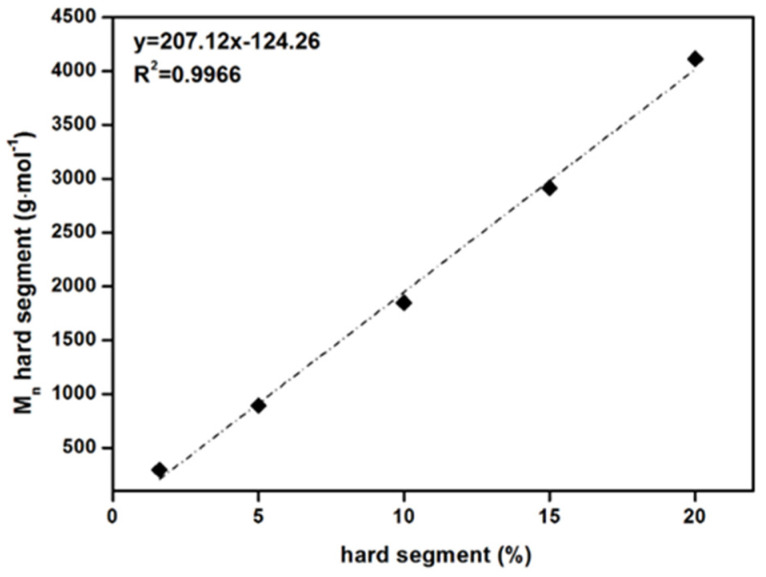

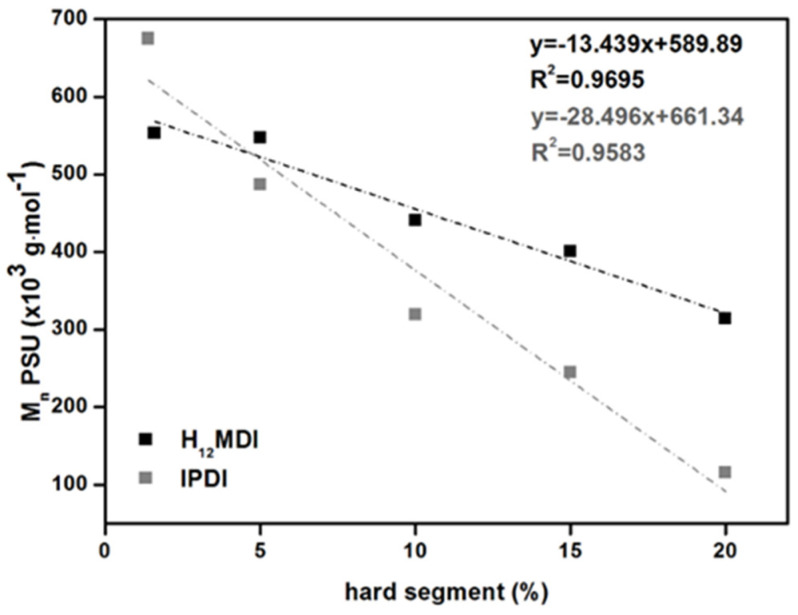
(**top**) Calculated average hard segment (HS) molecular weights as a function of HS content in PSU elastomers synthesized from H_12_MDI. (**bottom**) Number average molecular weights of PSU elastomers synthesized from H_12_MDI (black squares) and IPDI (grey squares) determined by SEC as a function of HS content.

As the amount of HS in the PSU elastomers increases from 1.5% to 20%, the molecular weight of the HSs increases linearly from approximately 300 to 4000 g·mol^−1^ (see Table 2 and Figure A1 in Appendix A). For the PSU copolymers synthesized without the chain extender, the structures consist of alternating blocks of diisocyanate and long PDMS chains.

Table 2 presents the molar ratios of the monomers used for the synthesis of PDMS-based polyurea elastomers, the molecular weights determined by SEC, and [*η*] values. Furthermore, the theoretical number average molecular weights (${\bar{M}}_{n}$) of the HSs, which were calculated according to the reaction stoichiometry, are also displayed.

All the PSU elastomers exhibit ${\bar{M}}_{n}$ values higher than 100,000 g·mol^−1^ and polydispersity indices (PDIs) between 2.0 and 2.5, except for the copolymer from MDI, which has a PDI close to 3.0. The ${\bar{M}}_{n}$ values of the PSU elastomers prepared from H_12_MDI and IPDI decrease almost linearly with increasing HS content. Conversely, the PSU copolymers and elastomers comprising an HS content of 5% exhibit very high number and weight average molecular weights (${\bar{M}}_{w}$). As expected, the values of [*η*] decrease from 1.16 to 0.32 dL·g^−1^ when the molecular weights of the PSU elastomers decrease. Interestingly, [*η*] seems to be sensitive to the HS content of the PSUs independently of HS structure. In a previous study concerning a series of PSU copolymers synthesized from H_12_MDI and PDMS with molecular weights between approximately 900 and 4000 g·mol^−1^, the values for intrinsic viscosity were 0.39, 0.45, 0.73, and 0.93 dL·g^−1^ for HS contents of 23%, 14%, 10%, and 6.5%, respectively [38]. These reported values are very close to the values determined here for the PSU IPDI elastomers, even though they are chain extended.

The differences in molecular weights of the PSUs might be attributed to the solubility of the HSs in the solvent used for synthesis of the elastomers, i.e., THF. The PSU copolymers were synthesized without a chain extender and, as a result, the HS molecular weights in these polymers are very low because the HS is exclusively formed by the diisocyanate and the terminal amino groups of the PDMS. Therefore, the local concentration of intermolecular hydrogen bonding between the urea groups is assumed to be low. Furthermore, the polar urea groups are separated from each other by long hydrophobic PDMS chains. Due to the dominantly hydrophobic nature of the PDMS, the copolymers dissolve well in THF. This probably explains the observed moderate viscosity of the polymer solution during synthesis, which may have allowed high-mobility monomers and hence high-molecular-weight polymers. However, when the HS content is increased, the HS chain length also increases because a large excess of diisocyanate reacts with the chain extender after formation of the prepolymer. Thus, long sequences of HSs are formed, leading to a high concentration of urea groups. As a result, the ability of the slightly polar solvent to interact with the strongly hydrogen-bonded urea groups is low. During synthesis of these elastomers, the chain extension step is accompanied by a pronounced increase in viscosity, which possibly reduces the mobility of the remaining chain extender molecules.

The observed differences in viscosity can be further explained in terms of the Hildebrand solubility parameters, which are 18.6 MPa^1/2^ for THF, 15.6 MPa^1/2^ for PDMS, and 45.6 MPa^1/2^ for the urea groups [21,39]. From these solubility parameters, one can deduce that THF preferentially dissolves the PDMS soft segments than the polar urea groups.

The synthesis of soluble and elastic PSU elastomers from the diisocyanates MDI and CHDI with HS contents exceeding 1.5% failed. Even solvent mixtures of 1:1 tetrahydrofuran/dimethylacetamide and 1:1 tetrahydrofuran/isopropanol were not polar enough to prevent gelation of the PSUs after addition of the chain extender. This behavior probably results from the formation of very strong hydrogen bonds between urea groups in the ordered HSs.

Versteegen et al. synthesized poly(THF)-based PUr copolymers with uniform HSs in order to study the effect of HS size and number of urea groups on the mechanical properties and processabilities of the resultant copolymers [40]. To obtain copolymers with 1–4 urea groups per HS unit, they applied a stepwise synthesis route and, in some cases, employed di-tert-butyl dicarbonate to partially protect the amino groups of the diamine used. Bis(3-aminopropyl)-terminated poly(THF) was either reacted directly with 1,4-diisocyanatobutane or was converted to an NCO-terminated prepolymer by reaction with di-tert-butyl tricarbonate followed by subsequent reaction with 1,4-diaminobutane. The copolymer, which contained two urea groups within the HS unit, exhibited good elastomeric properties and was both meltable and soluble in NMP. However, the copolymers containing three or four urea groups within the HS were neither meltable nor soluble and formed strong physically cross-linked gels. FTIR spectroscopic analysis of the copolymers revealed very strong hydrogen bonding interactions between the monodisperse HSs. Furthermore, they employed either 1,4-diisocyanatobutane or diaminobutane as spacer moieties between the urea groups, both of which are very symmetric due to the even number of methylene groups they contain. For instance, PUs containing 1,4-butane diol rather than 1,5-pentane diol as a chain extender exhibit superior mechanical properties because intermolecular hydrogen bonds are free of strain [41].

**Figure 2:**
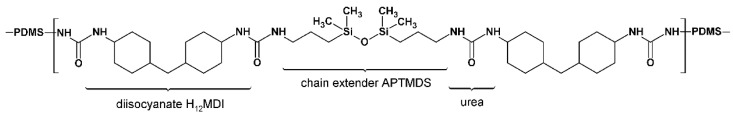
An average HS repeating unit. Example shown is that for the PSU elastomer based on H_12_MDI with an HS content of 5%. In this elastomer, the molar ratio of PDMS:NCO:APTMDS is approximately 1:2:1, represented in the structure by the PDMS followed by the HS repeating unit (within brackets) consisting of two diisocyanate molecules and one chain extender molecule. For reasons of simplification, the propyl-chains between the PDMS and urea groups are omitted from this structure.

In CHDI-based PUr copolymers, the intermolecular hydrogen bonds are planar, which allows very close packing of the HSs in PUs or PUrs. [31] Therefore, this result further confirms the gelling behavior observed during synthesis, particularly that of the CHDI-based PSU elastomers. As is apparent from Figure 2, an HS content of 5% leads to an average HS repeating unit containing four urea groups. However, increasing the HS contents in the PSU elastomers prepared from the diisocyanates H_12_MDI and IPDI does not lead to such solubility problems during synthesis. H_12_MDI is a mixture of three stereoisomers. The ratio of isomers is approximately 20% *trans-trans*-isomer, 50% *cis-trans*-isomer, and 30% *cis-cis*-isomer. IPDI is a mixture of two stereoisomers containing approximately 30% *trans*-isomer and 70% *cis*-isomer. As H_12_MDI and IPDI are composed of isomeric mixtures containing a low ratio of the symmetric *trans*-isomer, their HS structures are rather random [42,43].

### 3.2. Transparency of PDMS-Based PUr Elastomer Films

**Figure 3:**
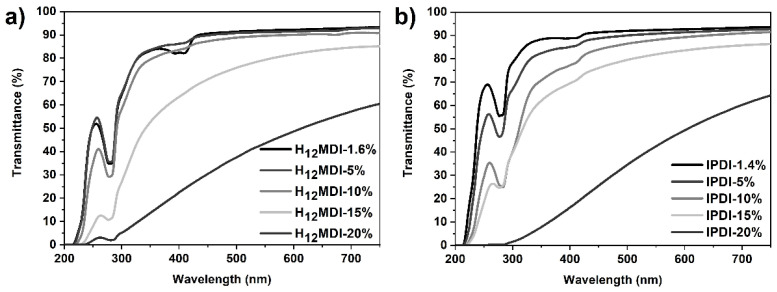
UV–Vis spectra of PSU elastomers synthesized from (**a**) H_12_MDI and (**b**) IPDI with HS contents between approximately 1.4% (copolymers) and 20%. All PSU elastomers are transparent until an HS content of 10% and exhibit transmittance values between approximately 91% and 93% at 750 nm. The transmittance of the PSU elastomers decreases to approximately 60% at an HS content of 20%.

**Figure 4:**
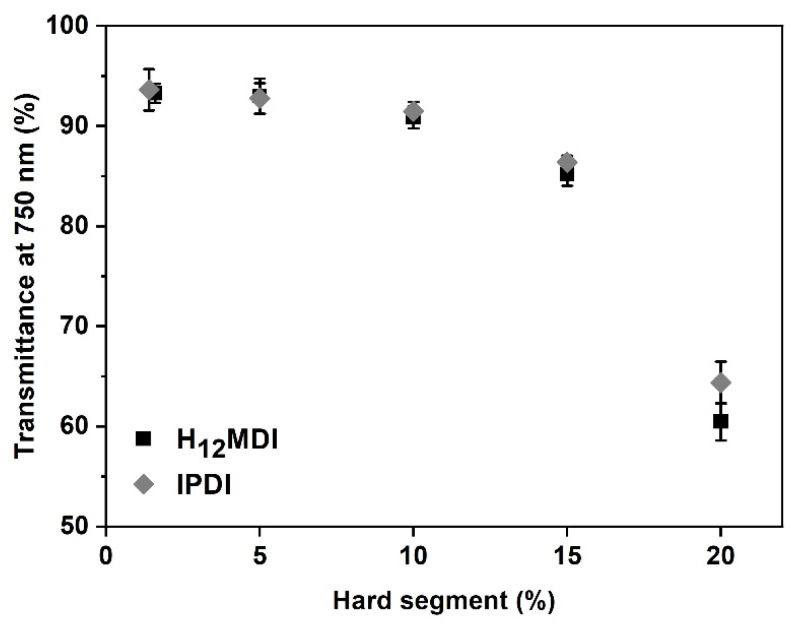
Influence of HS content on transmittance of PSU elastomer films synthesized from H_12_MDI and IPDI. Transmittance at 750 nm is higher than 90% in the PSU elastomers with HS contents between 1.4% and 10%. PSU elastomers with HSs contents of 15% and 20% are less translucent and have transmittance values of approximately 85% and 60%, respectively. The error bars represent the standard deviation from three measurements.

One of the objectives in this study was to study the influence of HS content and isocyanate structure on transparency. This is particularly important for PSU elastomers intended for application as biomaterials in optical applications, e.g., as intraocular lenses or soft contact lenses, where it is important that the materials exhibit a high transmittance within the visible light spectrum. As is apparent from Figure 3 and Figure 4, the PSU elastomers synthesized from H_12_MDI and IPDI are transparent for HS contents below 10% and exhibit transmittance values of 90% and higher at 750 nm. Notably, the spectra of both PSU copolymers reveal high transmittances of over 90% between 750 nm and approximately 450 nm, which is similar to that of a 20 diopters intraocular lens [44]. As the HS content increases to 15% and 20%, the PSU elastomers become increasingly translucent (see photographic images of PSU films in Figure A5 in Appendix D). The transmittances of the PSU films determined at 750 nm decrease to approximately 85% and 60% for HS contents of 15% and 20%, respectively.

Increasing the HS content in the PSU elastomers significantly affects the segmented structure of these polymers. At an HS content of 5%, the PSU elastomers consist of an alternating sequence of PDMS soft segments separated from each other by one HS repeating unit formed by the diisocyanate and the chain extender APTMDS (see Figure 2). As the HS content doubles from 5% to 10%, the sequence of PDMS soft segments and urea HSs within these elastomers is significantly altered. As is apparent from the molar ratios displayed in Table 2, approximately 4 mol of H_12_MDI react with 1 mol of PDMS in the prepolymer reaction. Thus, the remaining free diisocyanates react with 3 mol of APTMDS during the chain extension step, leading to a sequence of PDMS soft segments separated by three adjacent repeating HS units. As a result, the HS molecular weight also more than doubles from approximately 892 to 1848 g·mol^−1^. The HS chain length increases further when the HS content in the PSU elastomers is increased to 15% and 20%.

Due to the inherent incompatibility of the nonpolar soft segments with the polar HSs, PUrs, in particular, exhibit a very pronounced microphase separated morphology if no competitive hydrogen bonding between hard and soft segments occurs [40,45]. Unlike polyether-based soft segments, PDMS does not participate in intermolecular hydrogen bonding [45]. In these elastomers, urea groups form strong bidentate intermolecular hydrogen bonds, especially within long HS sequences. This leads to domains of well-ordered crystalline-like structures that scatter incident light if the aggregates are large enough. The example ATR-FTIR spectra in Figure A4 (see Appendix C) for the PSU H_12_MDI elastomers demonstrate that both N-H bonds and carbonyl groups are involved in ordered hydrogen bonding interactions [45].

In a previous study, in which the PDMS soft segment chain length was increased while the HS content of the PSU elastomers was kept constant at 10%, a comparable opacity was observed in the elastomers synthesized from PDMS with molecular weights of 23,000 g·mol^−1^ and higher [32]. The average HS molecular weight in this PSU elastomer was approximately 2600 g·mol^−1^, which is close to the HS molecular weight of 2900 g·mol^−1^ in the H_12_MDI-based elastomer with a 15% HS content. As the results of the present and previous studies show, the average HS molecular weight seems to be a critical factor for obtaining either optically clear (if ${\bar{M}}_{n\;\left(HS\right)}$ < 2200 g·mol^−1^) or translucent (if ${\bar{M}}_{n\;\left(HS\right)}$ > 2600 g·mol^−1^) PSU elastomer films.

**Figure 5:**
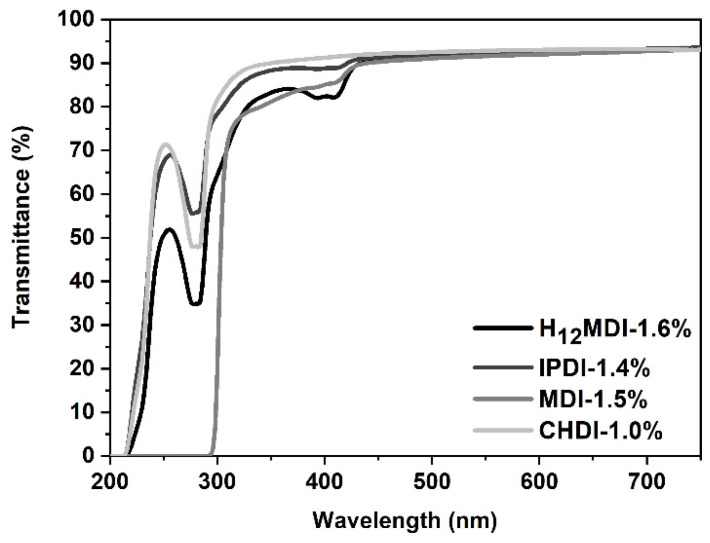
UV–Vis spectra of PSU copolymers synthesized from the aliphatic diisocyanates H_12_MDI, IPDI, and CHDI, and from the aromatic diisocyanate MDI without a chain extender. All PSU copolymers exhibit transmittance values of over 90% between 450 and 750 nm. Transmittance of the PSU copolymer film synthesized from MDI is zero below 300 nm owing to absorption of UV-B radiation by the aromatic rings.

Regardless of the diisocyanate used for synthesis, the transmittances of the PSU copolymers within the visible spectrum range (approximately 450–750 nm) are equal (see Figure 5). In the MDI-based copolymer, parts of the UV-B range are absorbed by the aromatic rings. When utilizing the PSU elastomers in optical applications such as contact or intraocular lenses, absorption of UV light is favorable because this protects sensitive eye structures and tissues such as the cornea, the crystalline lens, and the retina from the harmful impact of UV radiation. Therefore, the new generation of commercially available intraocular lenses contains chromophores and filters that selectively absorb parts of the blue and UV spectrum [46].

Interestingly, the MDI-based PSU copolymer does not show any yellowing upon exposure to UV light, which is a typical characteristic in aromatic PUs [47]. It is assumed that the urethane bridge in the aromatic PUs oxidizes to a quinone-imide structure upon exposure to UV light, which itself is a strong chromophore, leading to the yellowing of the polymer [47]. Most likely, the concentration of such structures is too low in the PSU copolymer.

### 3.3. Mechanical Properties

#### 3.3.1. Influence of HS Content on the Mechanical Properties of PDMS-Based PUr Elastomers

**Figure 6:**
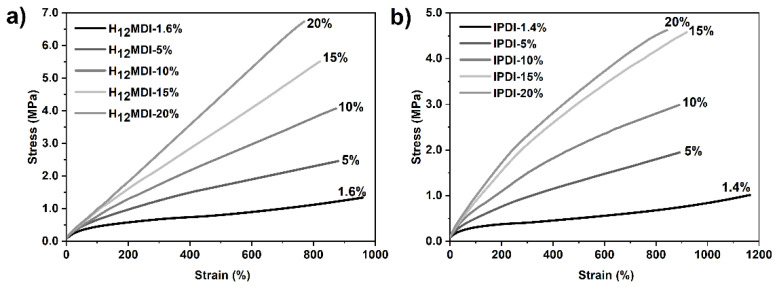
Representative stress–strain curves for PSU elastomers synthesized from (**a**) H_12_MDI and (**b**) IPDI with different HS contents. Young’s modulus (YM) for the PSUs increase with increasing HS content from approximately 1.4% to 20%, as is apparent by the steep increase of the slope within the linear ranges of the stress–strain curves. Regardless of their HS contents, all PSU elastomers are highly elastic and exhibited elongation at break values of over 800%.

As the representative stress–strain curves in Figure 6 show, the mechanical properties, particularly the YM and UTS values, of the PSU elastomers synthesized from H_12_MDI and IPDI are strongly dependent on their HS contents. In both PSU elastomer series, YM increases with the corresponding HS content from approximately 0.52 to 1.34 MPa in the H_12_MDI-based elastomers and from 0.27 to 1.28 MPa in the IPDI-based PSU elastomers.

**Figure 7:**
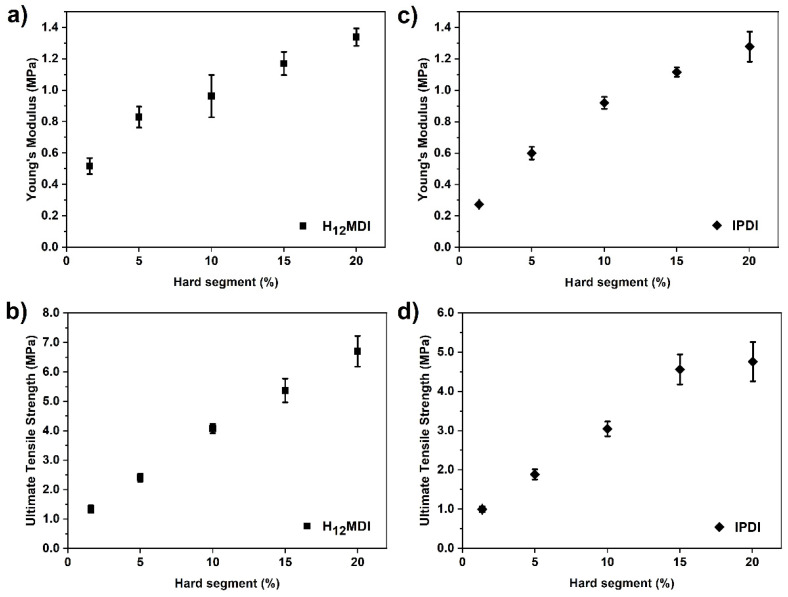
YM and ultimate tensile strength (UTS) values of PSU elastomer films synthesized from (**a**,**b**) H_12_MDI and (**c**,**d**) IPDI as a function of HS content. The values at approximately 1.6% and 1.4% HS content correspond to copolymers that were synthesized without chain extenders. The values between 5% and 20% HS content correspond to PSU elastomers that were chain-extended with APTMDS. The YM values of the PSU H_12_MDI-elastomers increases approximately linearly from 0.8 to 1.3 MPa. In the IPDI series, the values for YM increase logarithmically from approximately 0.6 to 1.3 MPa. UTS increases linearly with increasing HS content from 1.3 to 6.7 MPa (H_12_MDI) and from 1.0 to 4.6 MPa (IPDI). The value at 20% HS content in the IPDI series deviates slightly from linearity. The error bars represent the standard deviation from five measurements.

In Figure 7, the values of YM are plotted as a function of HS content. When regarding the series of chain-extended PSU elastomers, the values of YM seem to follow a linear trend in the H_12_MDI series and increase in a logarithmical manner in the IPDI series. Prisacariu and Scortanu reported that the YM of aromatic poly(ether urethane)s increases proportionally with HS contents [27]. Conversely, a logarithmic relationship of YM with increasing HS content was reported for copolymers based on PDMS-urea [38] and polyTHF-urea [48]. Gaymans et al. synthesized triblock urethane-urea-amide elastomers from PTMO-OH and PTMO prepolymers terminated with MDI and TDI, respectively, and applied the diamine-diamide chain extender N-aminohexyl-N-hexyl-terephthalamide (6T6) [49,50]. In these elastomers, the HS concentration was increased by increasing the PTMO soft segment length. In both series, a linear relationship between the logarithm of the storage modulus and HS content was observed [49,50]. Interestingly, a similar relationship was also reported for polyamides consisting of sebacic acid and hexamethylene diamine. When the amide hydrogens were partially substituted with isobutyl groups, the 100% modulus showed an approximately logarithmic trend toward lower values with increasing substitution degree [51]. These findings confirm the importance of intermolecular hydrogen bonding for the mechanical properties of amide, urethane, and urea block copolymers.

At low HS contents (1.4–5%), the PSU elastomers based on IPDI are softer than those synthesized from H_12_MDI, which is most probably an effect of the very asymmetric structure of this diisocyanate. As IPDI is a mixture of two isomers with different symmetries (*cis* and *trans*) containing three methyl groups and two chemically different isocyanate groups (primary and secondary), the intermolecular distances between the urea groups are not uniform [31]. Therefore, the strength of physical cross-linking within those PSU elastomers, which is important for the mechanical strength of the polymer, is low. However, at HS contents of 10% and higher, the values of YM in both PSU elastomer series are similar, which might be due to the increasing influence of urea concentration.

Values of UTS are displayed as a function of HS content in Figure 7b,d. In both series, UTS is directly proportional to HS content, and the values increase from 1.34 to 6.70 MPa (H_12_MDI series) and from 1.00 to 4.76 MPa (IPDI series). However, the value for UTS in the IPDI-based elastomer with 20% HS content deviates from the linear relationship by a factor of approximately 1.0 MPa.

The linear upward trend of tensile strength with increasing HS content (and thus with increasing concentration of urea groups) within the PSU elastomers confirms the findings of other authors. For instance, the Yilgör group conducted systematic studies on the relationship between morphology and mechanical properties in PDMS-based urea copolymers and block copolymers [22,38], poly(propylene oxide) (PPO)-based PUrs [52], and poly(isobutylene)-based urea block copolymers [53]. They reported that, regardless of the type of soft segment applied, a strong linear relationship between the HS content and tensile strength of the polymers is observed. Furthermore, the slopes of the obtained fitting curves did not seem to be greatly influenced by the symmetry of the applied chain extender but were affected by the molecular weight of the soft segment. This observation was explained in terms of a synergistic effect of entanglements arising in soft segments with high molecular weights [22]. A straight line is obtained when plotting stress-at-break against HS content of siloxane-containing poly(urea-oxamide) copolymers [54]. These results reveal that the nature and strength of the hydrogen bonds between the HSs play a major role in determining the extent of microphase separation in segmented PU and PUr elastomers [55]. As the large elongations of the PSU copolymers in Figure 6 and the values for mechanical performance (Figure 7) show, the bidentate urea hydrogen bonds are highly effective for providing sufficient mechanical strength combined with good elastomeric properties, even at very low HS contents.

#### 3.3.2. Influence of HS Content on Mechanical Hysteresis Behavior in PDMS-Based PUr Elastomers

Mechanical or tensile hysteresis commonly occurs in thermoplastic PU and PUr elastomers upon repeated loading and unloading cycles due to energy dissipation in the material [41,56]. In contrast to conventional rubber-like elastomers, which are covalently cross-linked and hence exhibit very low hysteresis values, segmented PUs and PUrs owe their mechanical stability and elasticity from a network of physical cross-links between urethane and urea groups, respectively, which are embedded in a matrix of amorphous or semi-crystalline soft segments. Upon applied strain, the microstructure of the hard domains is deformed or partially disrupted, leading to a loss of energy due to the conversion of mechanical energy to heat [42,57]. During the first loading cycle, the microphase organization within the polymer is markedly changed. As the consecutive relaxation and loading cycles generally proceed at the same speed, the polymer morphology cannot completely regain its initial structure within this short period. Therefore, the consecutive stress values reached at the same applied strain are generally lower leading to differences in hysteresis values between the first and subsequent cycles. This stress softening effect is characteristic for microphase separated polymers [58].

In our previous publication, we reported that hysteresis of PDMS-based PUr elastomers, all with an HS content of 10%, decreases when the molecular weight of the PDMS soft segment, and hence that of the HS, is increased [32]. Therefore, here we studied the effect of HS content on the hysteresis behavior of PSU elastomers prepared from PDMS with a constant molecular weight. In this study, a PDMS with a molecular weight of approximately 16,300 g·mol^−1^ was selected because the PSU elastomers we prepared previously from PDMSs with molecular weights between 15,000 and 18,000 g·mol^−1^ were transparent and had sufficient mechanical stability and low hysteresis values. When utilizing PDMS-based PUr elastomers in biomedical applications, such as wound dressings, vascular grafts, and intraocular lenses, it is crucial for their long-term performance that the materials do not undergo a large irreversible mechanical deformation upon repeated application of strain.

The 10-cycle hysteresis behavior was studied at two strains, 100% and 300%, because a previous study performed on a poly(ether urethane) revealed that the extent of hysteresis is not only sensitive to morphological changes but also increases with strain [57].

**Figure 8:**
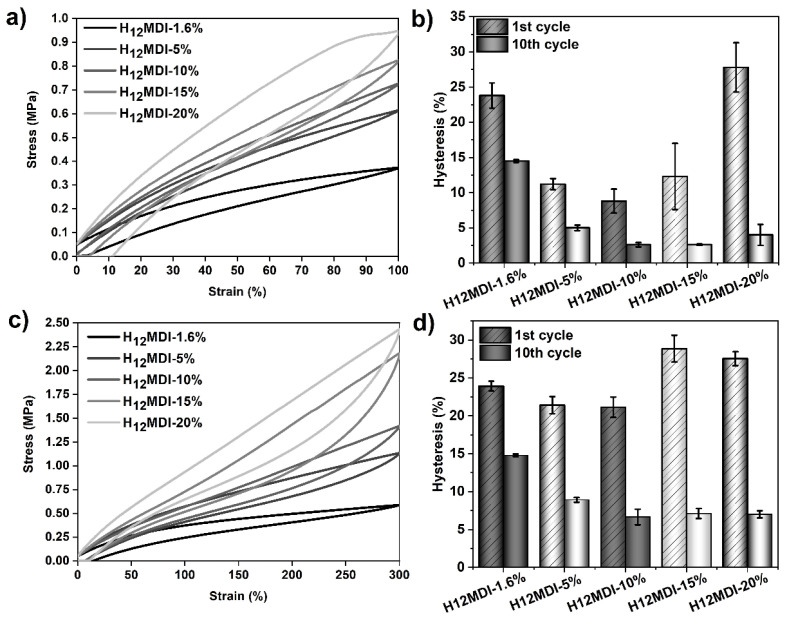
Results of tensile hysteresis measurements on PSU elastomers synthesized from H_12_MDI with HS contents between 1.6% (copolymer) and 20%. (**a**) 100%-hysteresis curves (1st cycle) and corresponding mean hysteresis values (**b**) of the 1st and 10th cycles. (**c**) 300%-hysteresis curves (1st cycle) and corresponding mean hysteresis values of the elastomers (**d**) at the 1st and 10th cycles. The error bars represent the standard deviation of three repeated measurements. Hysteresis at 100% strain in the 1st cycle decreases from approximately 24% (1.6% HS content) to 9% (10% HS content) and increases again to approximately 28% at 20% HS content. Hysteresis in the 10th cycle decreases from approximately 15% (1.6% HS content) to 3% (10% HS content) and remains constant with increasing HS content. A similar but less pronounced trend for the hysteresis values appears when the PSU elastomers are repeatedly stretched to 300%.

The values of tensile hysteresis, determined for the PSU elastomers based on H_12_MDI, are displayed in Figure 8 and Table 3. As expected, the hysteresis values in the first cycles are distinctly higher than those in the subsequent cycles, irrespective of applied strain. After the strain induced morphological reorganization of the HSs during the first stretch, the deformed HS domains remain largely unaffected by the applied strain in the following loading cycles, which is why the values decrease only very slightly or remain constant. Interestingly, the 100%-hysteresis values (1st cycle) of the PSU copolymer and the PSU elastomer containing 20% HS are quite similar. This leads to the assumption that hysteresis behavior is dependent on both the concentration of urea groups in the HS and the related hard domain morphology, i.e., randomly dispersed or interconnected.

When regarding the 100%-hysteresis values of the first cycles in this PSU series, it appears that hysteresis is determined by two different trends. At low HS contents (between 1.6% and 10%), hysteresis decreases from approximately 24% (H_12_MDI copolymer) to a minimum of 9% (PSU H_12_MDI-10%). At higher HS contents (15% and 20%) the hysteresis values increase from 12% to 28%, respectively. A similar trend is obvious for the values after the 10th cycle. However, for HS contents of 10% and higher, the hysteresis values remain constant.

Repeated cycling of the PSU elastomers at 300% strain presents a comparable, though less pronounced, trend of the hysteresis values in the first cycles. Hysteresis slightly decreases from 24% in the H_12_MDI copolymer to 21% in the PSU elastomers containing 5% and 10% HS. At HS contents higher than 10%, hysteresis increases again to approximately 29%. After the 10th cycle, hysteresis decreases from 15% (H_12_MDI copolymer) to 7% (PSU H_12_MDI-10%) and remains constant at higher HS contents.

Looking closer at the hysteresis values for the chain-extended PSU elastomers, it seems that changes in HS content between 10% and 20% do not significantly influence hysteresis behavior in the subsequent cycles as the values in each strain series (100% and 300%) are approximately equal. In other words, once the hard domains are deformed or partially disrupted by the breaking of inter-urea hydrogen bonds, which is most probably the case at high strains, the higher number of urea groups within the HSs obviously does not contribute to mechanical stabilization of the polymer morphology. Furthermore, when considering the unloading cycles of the PSUs with HS contents of 15% and 20%, it becomes apparent that the curves reach a stress value of zero before the elastomer is completely released from strain. This behavior, often referred to as “instantaneous set,” results from energy loss during plastic deformation of the sample [56]. This observation confirms that, particularly at 300% strain, some of the hydrogen bonds are disrupted because all the PSU elastomers exhibit a similar amount of set after the first and 10th cycle (see hysteresis curves of the 10th cycle in Figure A7 in Appendix E).

**Figure 9:**
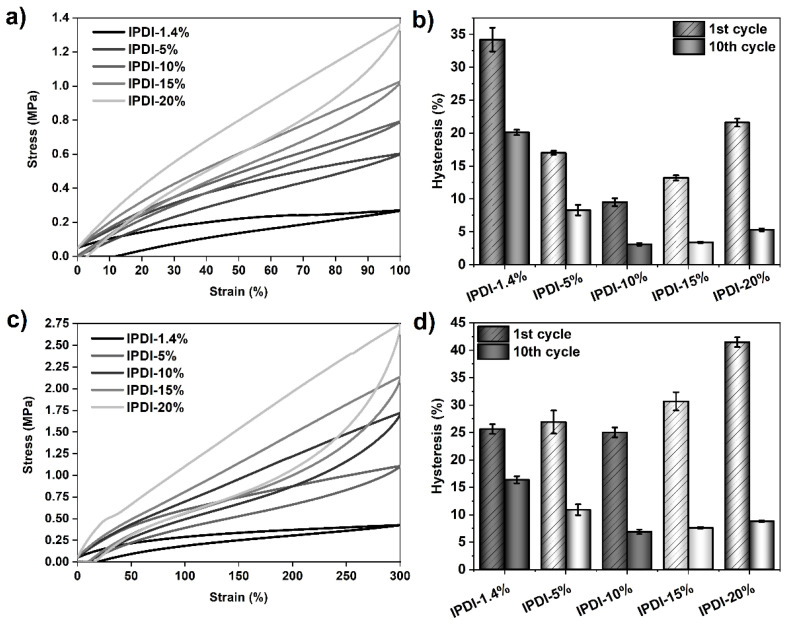
Results of tensile hysteresis measurements on PSU elastomers synthesized from IPDI with HS contents between 1.4% (copolymer) and 20%. (**a**) 100%-hysteresis curves (1st cycle) and corresponding mean hysteresis values (**b**) of the 1st and 10th cycles. (**c**) 300%-hysteresis curves (1st cycle) and corresponding mean hysteresis values of the elastomers (**d**) at the 1st and 10th cycles. The error bars represent the standard deviation of three repeated measurements. Hysteresis at 100% strain in the 1st cycle decreases from approximately 34% (1.4% HS content) to 10% (10% HS content) and increases again to approximately 22% at 20% HS content. Hysteresis in the 10th cycle decreases from 20% (1.4% HS content) to 3% (10% HS content) and slightly increases again to 5% at an HS content of 20%. At 300% strain, the lowest 1st-cycle-hysteresis value is measured in the PSU elastomer containing 10% HS. The highest hysteresis is over 41%, as observed for the elastomer with an HS content of 20%.

In contrast to the chain-extended PSU elastomers, where hysteresis is also dependent on the level of deformation, the hysteresis values in the H_12_MDI copolymer are equal irrespective of whether the copolymer is stretched to 100% or 300%. Similar behavior is also exhibited by the PSU copolymer synthesized from IPDI, albeit with most hysteresis values being slightly lower at 300% strain, as the graphs in Figure 9 and the values in Table 4 show. This indicates that the hydrogen bonds between single urea groups that are rather randomly dispersed within the PDMS matrix are weak and as a result are easily disrupted at a low strain level. Moreover, both copolymers displayed a set in the first cycle of the 100% hysteresis curves, while the PSU elastomers containing four and eight urea groups within one HS repeating unit did not exhibit this behavior.

The effect of HS content on the 10-cycle hysteresis behavior was additionally studied in an analogous series of PSU elastomers containing the diisocyanate IPDI. As the values in Figure 9 and Table 4 show, a similar trend for hysteresis behavior to that of the H_12_MDI series is observed. 100%-hysteresis in the 1st cycle decreases from 34% (IPDI copolymer) to a minimum of 10% in the PSU elastomer containing 10% HS. Upon further increase of the HS content from 15% to 20%, hysteresis increases from 13% to approximately 22%, respectively. After the 10th cycle, hysteresis decreases dramatically from 20% (IPDI copolymer) to 3% in the IPDI elastomers containing 10% and 15% HS and slightly increases again to 5% in the PSU IPDI-20% elastomer.

As already shown for the PSU H_12_MDI series, the trend in hysteresis at 300% strain is also less pronounced in the PSU IPDI series. Hysteresis in the IPDI-based elastomers, particularly at low HS contents, is higher than in the corresponding elastomers containing H_12_MDI. This may be attributed to the effect of less-ordered hydrogen bonds within small particulate hard domains. However, at HS contents of 10% and higher, this supposed symmetry effect may not be valid as the values of hysteresis in the first and last cycle are approximately equal in both the H_12_MDI and IPDI series. Overall, the highest obtained hysteresis is 41.5%, as observed in the PSU IPDI-20% elastomer.

The trend of the first-cycle values obtained for both PSU series between 1.6% (1.4%) and 10% HS content is contrary to the hysteresis results reported by Yilgör et al., who synthesized PDMS-based PUr elastomers from H_12_MDI and the chain extenders 1,2 ethylenediamine and 2-methyl-1,5-diaminopentane [56]. His group studied the effect of PDMS molecular weight, chain extender structure, and HS content on the 200%- and 300%-hysteresis behavior of PSU elastomers. For a series of elastomers synthesized from PDMS with a molecular weight of 31,500 g·mol^−1^, they reported that first-cycle hysteresis values (300% strain) increased linearly from 12% to 26% when the HS content increased slightly from 2.8% to 4.7%. However, in another series of PSUs synthesized from a PDMS of approximately 11,000 g·mol^−1^ with HS contents between 7.8% and 11.4%, which corresponds to an equal increase of HS molecular weight, the 200% hysteresis values did not increase significantly [21,56].

This clearly indicates that the HS content and its molecular weight in the PSU elastomer do not determine the hysteresis behavior exclusively. Furthermore, other factors such as soft segment molecular weight and nature of the HSs (crystallizing or not crystallizing) have a major influence on microphase morphology and hence on mechanical behavior [59]. In particular, 1,2-ethylenediamine is a very short and symmetric chain extender, thus promoting the formation of ordered and crystalline HSs. Conversely, the disiloxane-based chain extender applied in this study is very flexible owing to the large Si-O-Si bond angle (145–150° in hexamethyldisiloxane) and rather mimics the properties of the soft segment [60]. For instance, Adhikari et al. reported that incorporation of 40 mol% of the disiloxane-based chain extenders 1,3-bis(4-hydroxybutyl) and (3-hydroxypropyl)-tetramethyldisiloxane along with 1,4-butanediol increased the solubilities of the HSs in the PDMS/PHMO soft phases [61,62]. Yildirim et al. simulated the morphology of segmented aliphatic silicone-urea copolymers with varying HS contents using DPD calculations [63]. Models for PSU copolymers revealed a microphase separated morphology even at the very low HS content of 1.7%. When the HS content was increased by decreasing the PDMS molecular weight, the morphology changed from spherical randomly dispersed hard domains to large ribbon-like crystalline hard domains that became interpenetrating, particularly at high urea HS contents [63].

Therefore, in the present study we assumed that the PSU morphology consists of small randomly distributed hard domains surrounded by a continuous phase of PDMS at low HS contents between 1.4% and 5%. Even though the concentration of urea groups within the HSs is low, they clearly mechanically stabilize the soft segment chains by intermolecular hydrogen bonds, which act as a reinforcing filler. As a result, the hysteresis in the PSU elastomers is decreased. At an HS content of 10%, the hard domains are larger but presumably still discrete. However, when the HS content is increased further to 15% and 20%, the high concentration of urea groups within the HS repeating units leads to large thread-like hard domains with some crystallinity. This may also explain why the transparency of these PSU films decreases with increasing HS content. Such large hard domains are expected to be less flexible under mechanical stress, leading to disruption of the hydrogen bonds, even at low strains of 100%.

Abouzahr et al. investigated the structure property relationship for poly(ether urethane)s containing HSs formed by MDI and 1,4-butanediol with contents between 15% and 45% [64]. Their SAXS results revealed that the morphologies of the polymers changed with increasing HS content. At an HS content of 15%, some of the hard domains were solubilized in the soft phase. Isolated hard domains were found in the polymer with a 25% HS content. This PU exhibited good elastomeric properties and low hysteresis. At high HS contents (35–45%), the hard domains became interlocking and, as a result, hysteresis in those PUs was very high. This observation was explained in terms of an irreversible restructuring of the hard domain morphology. Furthermore, it was observed that disruption of the hard domains began at low elongations.

Wang et al. made similar observations for poly(ether urethane urea)s with HS contents between 36% and 46% containing 1,2-ethylenediamine [65]. They also observed high initial hysteresis at low strains in those polymers. In addition, they reported that the concentration of hydrogen bonded urea groups decreases after mechanical deformation.

#### 3.3.3. Influence of Diisocyanate Structure on the Mechanical Properties and Hysteresis Behavior of PSU Copolymers

The mechanical properties and hysteresis behavior of a series of PSU copolymers based on diisocyanates with different symmetries synthesized without a chain extender was investigated.

The ability of the urea groups to form strong intermolecular hydrogen bonds largely depends on the order of aligned HSs, which in turn determines the density of packed chains. The degree of microphase separation in PUs and PUrs generally increases if they contain a high concentration of hydrogen bonded urethane or urea groups and if they exhibit a distinct domain structure comprising crystalline hard and amorphous soft segments. Therefore, we selected the highly symmetric CHDI and compared the results of its tensile and hysteresis measurements with those for PSU copolymers based on the asymmetric IPDI and the diisocyanates H_12_MDI and MDI.

**Figure 10:**
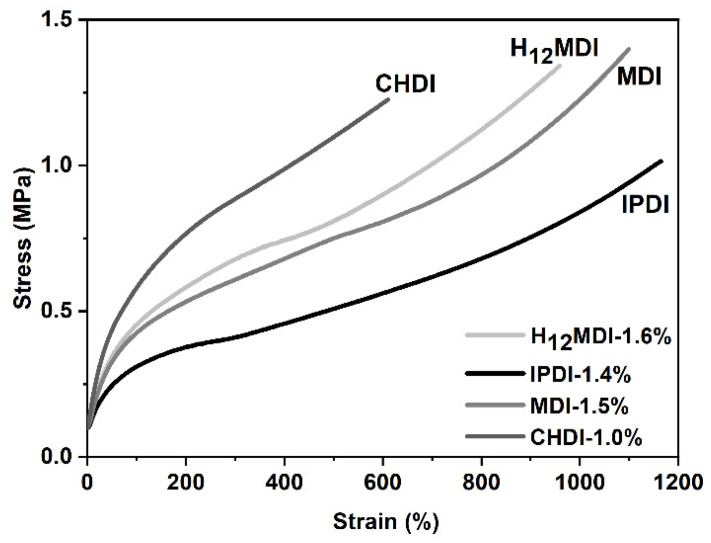
Representative stress–strain curves of PSU copolymers synthesized from the aliphatic diisocyanates H_12_MDI, IPDI, and CHDI and the aromatic diisocyanate MDI without chain extenders. The YM is highest for the CHDI-based copolymer, as is apparent from the steep increase of stress within the linear range of the stress–strain curves. The stress–strain curves of the copolymers based on H_12_MDI and MDI present approximately equal slopes and exhibit elongations of more than 800%. The lowest YM value is presented by the curve of the IPDI-based copolymer.

**Figure 11:**
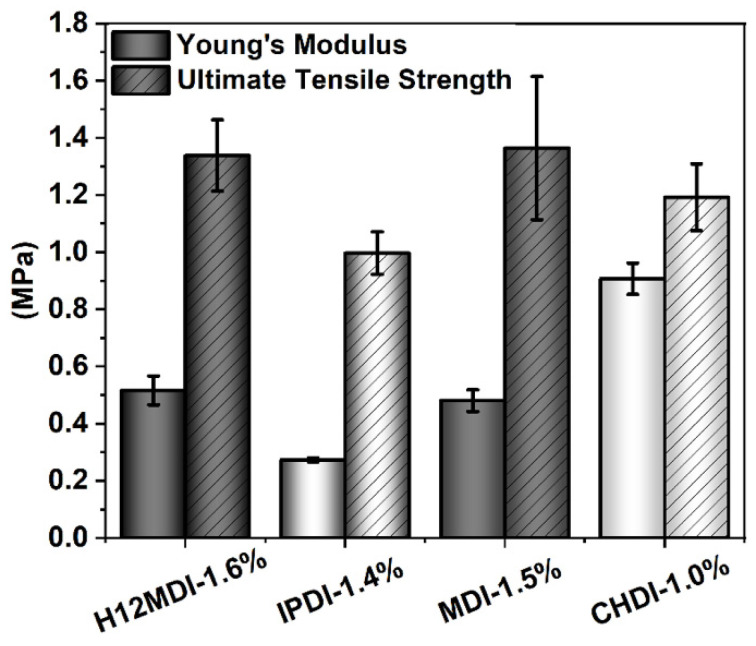
YM (left columns) and UTS (striped-columns) values for PSU copolymers synthesized from the aliphatic diisocyanates H_12_MDI, IPDI, and CHDI, and from the aromatic diisocyanate MDI without chain extenders. The error bars represent the standard deviation from five repeated measurements. YM is lowest for the unsymmetric diisocyanate IPDI and highest for the very symmetric diisocyanate CHDI. The YM and UTS values are approximately equal for the PSU copolymers synthesized from H_12_MDI and MDI.

The representative stress–strain curves of the copolymers are displayed in Figure 10. As is apparent from the slopes of the stress–strain curves and the mechanical properties shown in Figure 11, the copolymers based on H_12_MDI and MDI both exhibit YM values of ca. 0.5 MPa. Not surprisingly, the YM of the CHDI-based copolymer is highest in this series and almost twice those of the copolymers based on H_12_MDI and MDI. Conversely, the IPDI-based copolymer exhibits a YM of approximately 0.3 MPa, the lowest in this copolymer series.

**Figure 12:**
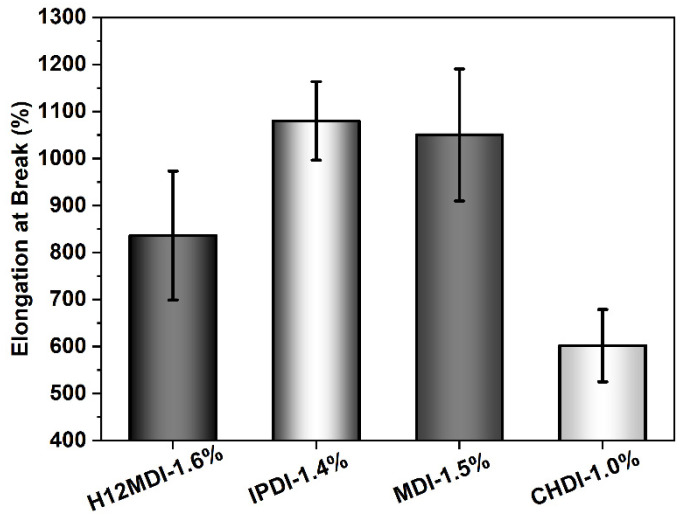
Elongation at break values for PSU copolymers synthesized from the aliphatic diisocyanates H_12_MDI, IPDI, and CHDI, and from the aromatic diisocyanate MDI without chain extenders. The error bars represent the standard deviation from five repeated measurements. Except for the CHDI-copolymer, which exhibits an elongation of approximately 600%, all the PSU copolymers are highly elastic with elongations between 800% and 1100%.

An approximately similar trend is observed for the UTSs. The PSU copolymers based on CHDI, H_12_MDI, and MDI exhibit similar UTS values, i.e., between 1.2 and 1.4 MPa. The lowest UTS of 1.0 MPa is observed for the IPDI-based copolymer. One would expect that the CHDI-based copolymer would also have the highest UTS because its HSs are presumably highly ordered. However, as this elastomer is stiffer than all the others in this series, its elasticity as reflected by its elongation at break is significantly lower (Figure 12). Conversely, the elastomers based on MDI and IPDI are highly elastic and present elongation values of 1050% and 1080%.

In the previous subsection, in which hysteresis behavior was investigated as function of HS content, it became obvious that a slight increase in HS content led to a mechanical stabilization of the physically cross-linked polymer network. As a result, the hysteresis values decreased and reached a minimum at 10% HS content. Conversely, higher urea concentrations made the HSs increasingly prone to deformation or disruption of the intermolecular hydrogen bonds upon applied strain. As the copolymers were solely synthesized from the diisocyanate and the amino-terminated PDMS, the HS contents and their molecular weights were low. Hence, the extent of hysteresis was expected to be dependent only on the degree of ordered HSs, which is in turn governed by the symmetry of the diisocyanate.

**Figure 13:**
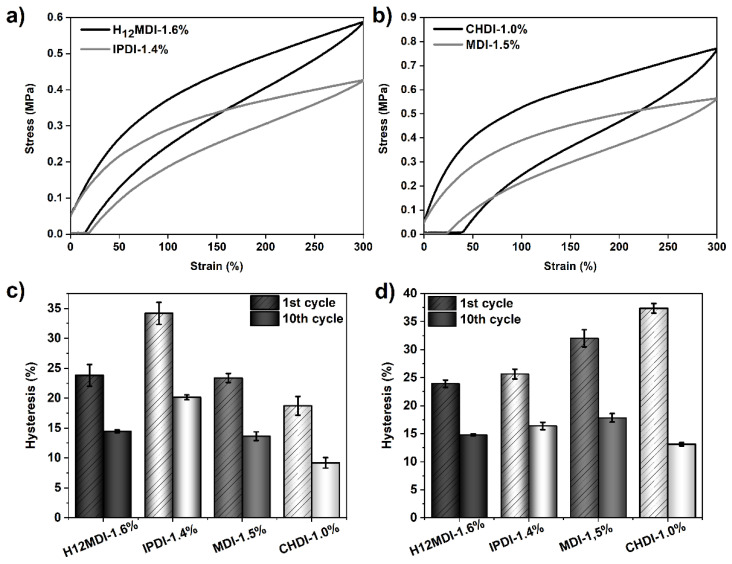
Results of tensile hysteresis measurements for PSU copolymers synthesized from the aliphatic diisocyanates H_12_MDI, IPDI, and CHDI, and from the aromatic diisocyanate MDI without chain extenders. 300%-hysteresis curves (1st cycle) of the copolymers based on (**a**) H_12_MDI and IPDI and (**b**) CHDI and MDI. Mean hysteresis values of the copolymers at the 1st and 10th cycle at (**c**) 100% strain and (**d**) 300% strain. The error bars represent the standard deviation of three repeated measurements. At 100% strain, the highest hysteresis is observed for the IPDI-based copolymer. At 300% strain, the MDI- and CHDI-based copolymers exhibit the highest hysteresis values.

Supposing that the HS order is highest in the CHDI-based elastomer and lowest in the IPDI-based elastomer, one would expect to observe the lowest hysteresis for the CHDI-elastomer because hydrogen bonds in those HSs are free of strain and therefore very strong. This assumption seems to be true in the range of low strains, where indeed the mean hysteresis in the first cycle is 19% for the CHDI-based elastomer and 34% for the elastomer synthesized from IPDI, as the bars in Figure 13c show. A clear difference in hysteresis behavior between the CHDI- and IPDI-based elastomers is apparent after the 10th cycle. As the values in Table 5 show, hysteresis for the elastomer containing CHDI decreases to 9% at 100% strain and to 13% at 300% strain. However, hysteresis in the IPDI-based elastomer remains high at 16% and 20%.

When the PSU copolymers are elongated to 300%, another situation is apparent. The highest first-cycle hysteresis values are observed in the elastomers based on CHDI (37%) and MDI (32%) as the graphs in Figure 13d show. Furthermore, both elastomers show a higher instantaneous set, which indicates more effective hydrogen bonding within the hard domains owing to the higher order of the HSs [28]. However, the hysteresis values after the third cycle become quite similar, irrespective of diisocyanate structure. From the different hysteresis behavior of the copolymer containing the symmetric CHDI, it can be concluded that the ordered HSs are more densely packed due to strong hydrogen bonding interactions. This seems to partially protect the morphology from deformation at low strains. At high strains, however, the morphology of the crystalline hard domains is disrupted, leading to a pronounced increase in hysteresis.

Das et al. synthesized several PTMO-based non-chain-extended PUs und PUrs from diisocyanates of varying symmetry and studied their morphologies and mechanical properties [28]. Unlike the PU copolymers, all the urea copolymers displayed well-separated microphase morphologies with thread-like hard domains and showed high mechanical strengths combined with good elastomeric properties. When comparing the results of the first-cycle hysteresis at 300% strain, they found that the values increased in the order H_12_MDI < MDI < CHDI. Furthermore, the instantaneous set was found to increase in the same order and was highest in the copolymer based on the symmetrical CHDI due to the breaking and restructuring of the crystalline hard domains [28]. A similar symmetry effect on the first-cycle hysteresis was reported by Prisacariu and Scortanu, who prepared polyether-based PUs from MDI and crystallizable DBDI [27].

### 3.4. In Vitro Cytotoxicity

All PSU elastomers and copolymers were subjected to in vitro cytotoxicity assessment to determine whether these polymers, which were synthesized from different diisocyanates with varying HS contents, have adverse effects on HaCaT cell viability. An aliphatic polycarbonate-based medical-grade PU (Carbothane PS 3585A) was selected as non-cytotoxic reference material.

**Figure 14:**
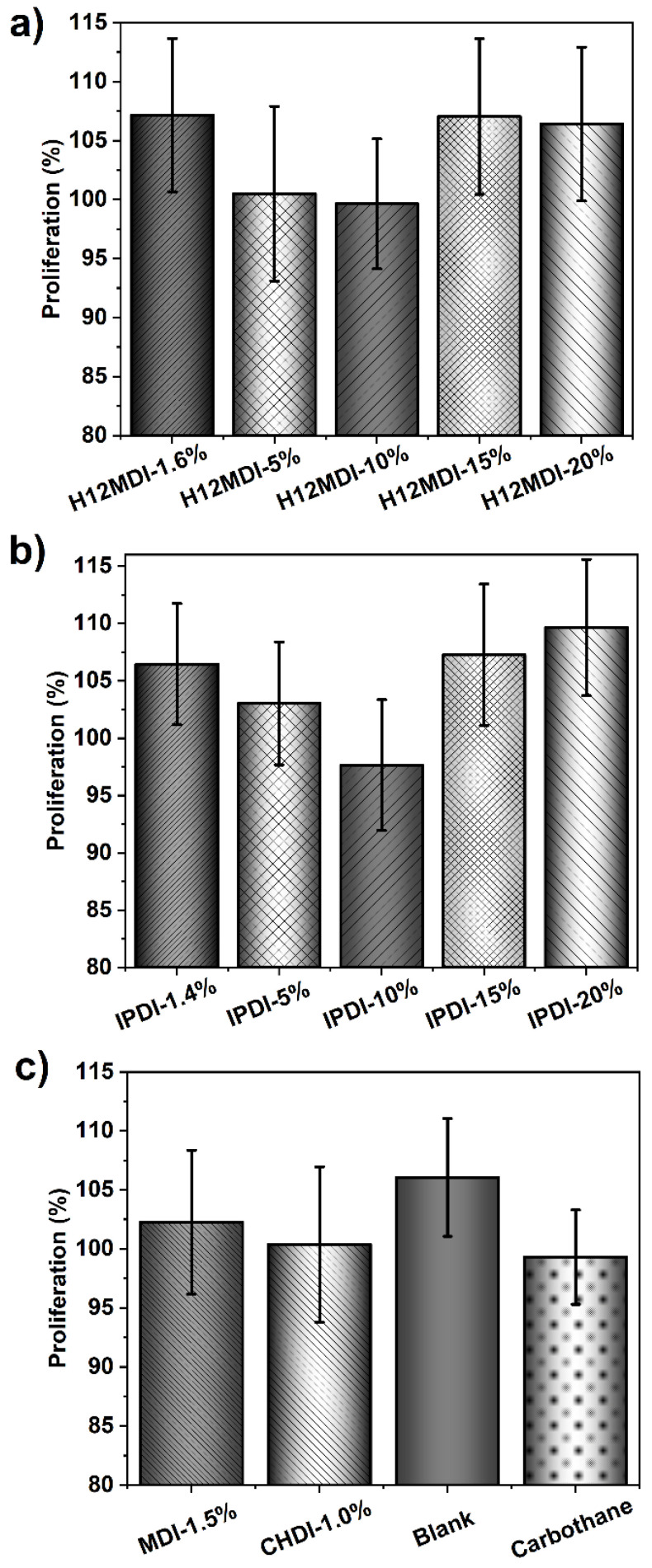
Viability of HaCaT cells after treatment with cell-medium extracts of PSU elastomers synthesized from (**a**) H_12_MDI, (**b**) IPDI, and (**c**) PSU copolymers synthesized from MDI and CHDI without chain extenders. The blank represents cell medium without a sample subjected to the same extraction conditions as the PSU samples. A medical-grade PU (Carbothane PS-3585A) was chosen as a reference material. Cell viabilities, as determined using the MTS cell proliferation assay, for all PSUs, PSU copolymers, and Carbothane are all over 95%. The error bars represent the standard deviation from 18 repeated measurements for each polymer-sample. Thus, no polymer extracts show any cytotoxic effects.

As the cell proliferation results in Figure 14 show, the cell-medium extracts of the PSU elastomers and copolymers clearly do not contain any cytotoxic low-molecular-weight leachables. All the PSU extracts exhibit a cell proliferation of over 97% and thus are not cytotoxic. These results confirm that it is beneficial to apply amino-terminated monomers to the synthesis of elastomers because the high reactivity of these groups toward aliphatic diisocyanates prevents the formation of low-molecular-weight chains and, more importantly, ensures complete conversion of the isocyanate groups without the use of toxic organotin-based catalysts. As expected, the reference material Carbothane also shows no cytotoxic effects.

## 4. Conclusions

Three series of PDMS-based urea elastomers were prepared by two-step polyaddition reactions in THF solution. The main objectives were to investigate the influence of (i) HS content and (ii) diisocyanate chemical structure (H_12_MDI, IPDI, MDI, and CHDI) on the transparencies and mechanical properties of the resultant PSU elastomers. Particularly, the effects of altered hard domain morphology upon increasing the content of urea groups on YM, UTS, and 10-cycle hysteresis behavior was interesting. For this reason, the HS contents in the PSUs based on IPDI and H_12_MDI were increased stepwise from 5% to 20% through incorporation of an increasing ratio of the diisocyanate and the chain extender APTMDS. Attempts to similarly increase the HS contents in the MDI- and CHDI-based elastomers resulted in tough and opaque polymer gels that were insoluble even in polar solvents and solvent mixtures.

Furthermore, the effects of diisocyanate structure on transparency and mechanical properties were studied on a series of non-chain-extended PSU copolymers, which were prepared with very low urea HS contents of 1.0–1.6%.

Transparent PSU elastomers are obtained for HS contents below 10%. Increasing opacity in the PSU films is observed for HS contents of 15% and higher. Transmittance at 750 nm, which is the upper edge of the visible light region, decreases to 85% and 60% at HS contents of 15% and 20%, respectively. This loss in transparency is attributed to light scattering by large thread-like hard domains that possibly form due to an increase in the HS molecular weight from 2900 to 4100 g·mol^−1^.

Independently of the diisocyanate structure and hence of the structure of the HSs, all the PSU copolymers exhibit transmittances of over 90% between 750 and 450 nm, making them potentially suitable for optical applications such as soft contact lenses or intraocular lenses. Furthermore, the MDI-based copolymer shows a cut off at approximately 300 nm and, as a result, blocks UV-B radiation. This may be a beneficial property in applications where UV sensitive materials or biological structures need to be protected. Interestingly, though this elastomer contains an aromatic diisocyanate, the typical phenomenon of yellowing upon exposure to UV light is not observed for this elastomer.

There is a linear correlation of YM and UTS with HS content in the PSU elastomers based on H_12_MDI. Conversely, values of YM in the IPDI series follow a logarithmical trend with increasing HS content. YM is highest for the CHDI-based copolymer due to the high symmetry of the diisocyanate, which allows a highly ordered and tightly packed HS structure. Conversely, the elastomer based on the asymmetric IPDI exhibits the lowest YM. The PSU copolymers based on H_12_MDI and MDI exhibit intermediate and similar YM values. All PSUs (except CHDI) exhibit high elasticity, displaying elongations between 760% and 1100%.

The hysteresis values for the first cycle exhibited two different trends. At low HS contents (between 1.4% and 10%), an increasing concentration of urea groups mechanically stabilizes the elastomer from the impact of strain, resulting in a reduction of hysteresis. It is presumed that the morphology in these elastomers consists of small, randomly distributed hard domains that physically reinforce the continuous PDMS soft phase by inter-urea hydrogen bonding. However, when the HS content is increased from 10% to 20%, the first cycle hysteresis values increase. This effect was explained in terms of an altered hard domain morphology. Due to an increase in HS molecular weight, it is postulated that the hard domains increase in size and presumably form a more interpenetrating network of thread-like hard domains. Upon the impact of strain, these domains are distinctly deformed or disrupted, leading to a higher hysteresis in the first cycle. However, the hysteresis values remain nearly constant after the 3rd cycle, whether the HS content is 10% or more. As expected, a higher strain (300% vs. 100%) induces higher hysteresis values in the PSU elastomers and in the copolymers based on MDI and CHDI. Interestingly, the hysteresis values of the H_12_MDI-copolymer are equal for both strains and, in the case of IPDI, slightly reduced. This may indicate that monodisperse urea HSs formed by the unsymmetric diisocyanates were already disrupted at low strain.

When comparing the results of mechanical characterization of all PSUs, an optimum balance between reasonable mechanical stability, elasticity, and low hysteresis is identified for the elastomers PSU H_12_MDI-10% and PSU CHDI-1.0%.

None of the PSUs exhibit any cytotoxic effects on HaCaT cells, demonstrating their potential for use in biomedical applications.

## Figures and Tables

**Table 1:** Molecular weights of the aminopropyl-terminated PDMSs used for synthesis of PSU elastomers in this study.

PDMS ^1^	Molar Ratio (D_4_/APTMDS)	Conversion (%)	${\bar{M}}_{n}$ Theoretical ^2^ (g·mol^−1^)	${\bar{M}}_{n}$ Titration ^3^ (g·mol^−1^)
PDMS (H_12_MDI/IPDI)	60.6/1.0	88.8	16,175	16,338
PDMS (MDI)	60.5/1.0	88.0	16,013	16,296
PDMS (CHDI)	60.6/1.0	89.5	16,295	16,472

^1^ PDMS was reacted with the diisocyanate displayed within the brackets. ^2^ Theoretical ${\bar{M}}_{n}$ was calculated according to ${\bar{M}}_{n}=(\mathrm{conversion}/100\%)\cdot \frac{m\left(D_{4}\right)+m(\mathrm{APTMDS})}{n(\mathrm{APTMDS})}$. ^3^ ${\bar{M}}_{n}$ was obtained as an average value from four titrations. PDMSs: poly(dimethylsiloxanes); PSU: PDMS-based PUr elastomers; D_4_: octamethylcyclotetrasiloxane; APTMDS: 1,3-bis(3-aminopropyl)-tetramethyldisiloxane; H_12_MDI: 4,4′-methylenebis(cyclohexyl isocyanate); IPDI: isophorone diisocyanate; MDI: 4,4′-methylenebis(phenyl isocyanate); CHDI: *trans*-1,4-cyclohexane diisocyanate.

**Table 2:** Molecular weights of the aminopropyl-terminated PDMSs used for synthesis of PSU elastomers in this study.

PSU	Molar Ratio (PDMS/NCO/CE)	${\bar{M}}_{n}$ SEC (×10^3^ g·mol^−1^)	${\bar{M}}_{w}$ SEC (×10^3^ g·mol^−1^)	PDI	Intrinsic Viscosity [*η*] (dL·g^−1^) ^1^	${\bar{M}}_{n}$ HS ^2^ (g·mol^−1^)
PSU H_12_MDI-1.6% *	1.0/1.0/0.0	553	1185	2.14	1.05	295
PSU H_12_MDI-5%	1.0/2.2/1.2	547	1314	2.33	0.77	892
PSU H_12_MDI-10%	1.0/4.0/3.0	441	859	1.95	n.a ^#^	1848
PSU H_12_MDI-15%	1.0/6.1/5.1	401	832	2.07	n.a ^#^	2915
PSU H_12_MDI-20%	1.0/8.5/7.5	314	707	2.26	n.a ^#^	4116
PSU IPDI-1.4% *	1.0/1.0/0.0	675	1498	2.22	1.06	257
PSU IPDI-5%	1.0/2.4/1.4	487	1046	2.15	0.86	892
PSU IPDI-10%	1.0/4.4/3.4	319	631	1.98	0.65	1849
PSU IPDI-15%	1.0/6.7/5.7	245	506	2.06	0.48	2916
PSU IPDI-20%	1.0/9.2/8.2	116	288	2.48	0.32	4126
PSU MDI-1.5% *	1.0/1.0/0.0	635	1853	2.92	1.16	285
PSU CHDI-1.0% *	1.0/1.0/0.0	403	829	2.06	0.77	194

**Figure A2:**
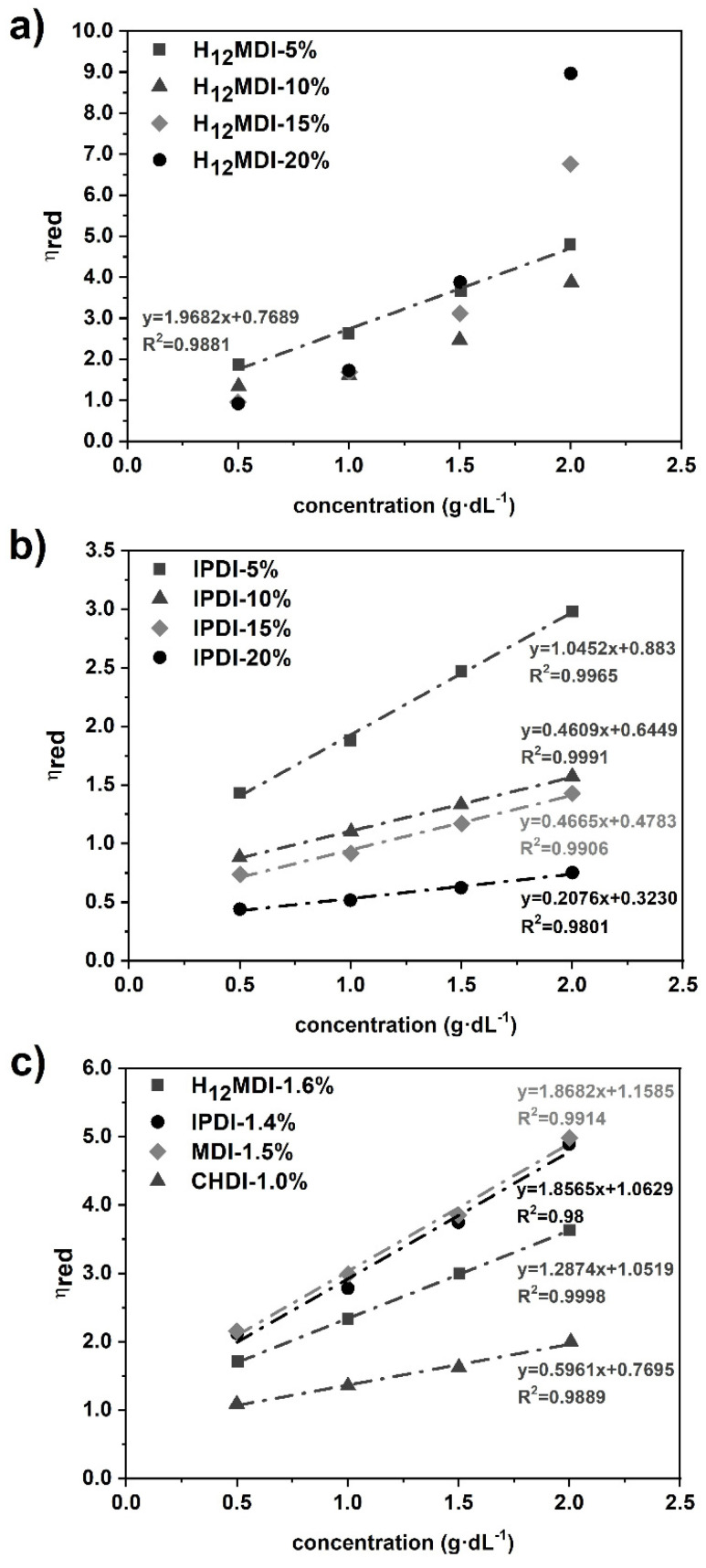
Huggins plots of reduced viscosity (*η*_red._) versus concentration (g·dL^−1^) of four PSU solutions in THF. The intrinsic viscosity [*η*] was calculated by extrapolation of the Huggins fitting curves. (**a**) Huggins plots of the PSU H_12_MDI elastomers comprising HS contents between 5% and 20%. A linear fitting curve was only obtained for the elastomer comprising 5% HS content. Values of reduced viscosity of the PSU H_12_MDI elastomers containing higher HS contents behaved not linear and seemed to rather follow an exponential trend. (**b**) Huggins plots of the PSU IPDI elastomers containing HS contents between 5% and 20%. (**c**) Huggins plots of PSU copolymers that were synthesized without chain extender.

**Figure A4:**
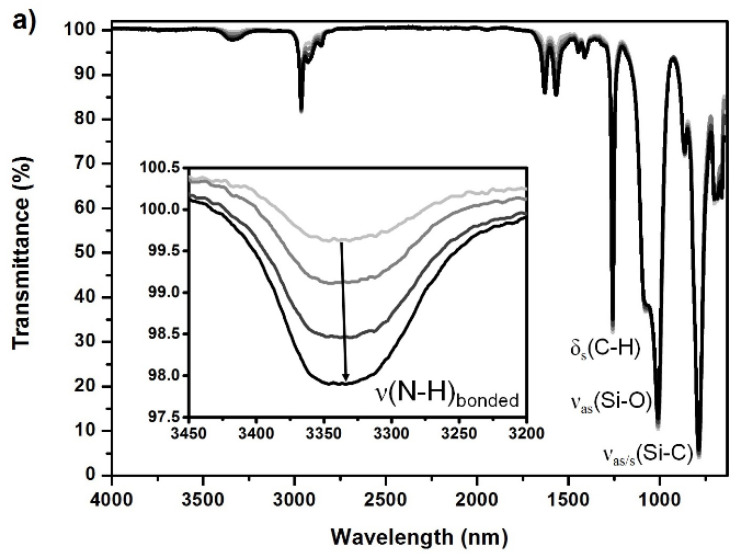

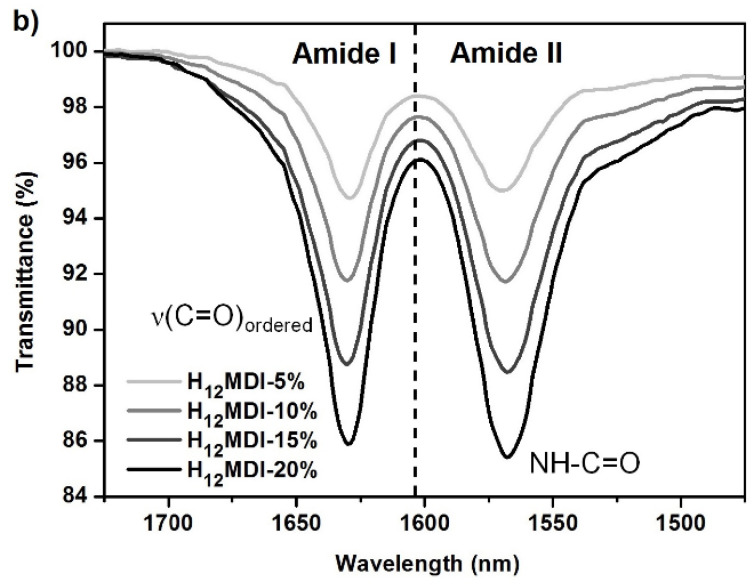
ATR-FTIR spectra of PDMS-based polyurea elastomers synthesized from H_12_MDI with increasing HS contents (5–20%). (**a**) The enlarged N-H stretching region at 3450–3200 cm^−1^ shows that all urea groups were involved in hydrogen bonding interactions. The bands below 1500 cm^−1^ correspond to the PDMS soft segment vibrations at: 1258 cm^−1^ (δ_s_ C-H in CH_3_), 1010 cm^−1^ (ν_as_ Si-O), 864 cm^−1^ (δ_as_ C-H in Si-(CH_3_)_2_), and 786 cm^−1^/702 cm^−1^ (ν_as/s_ Si-C in Si-(CH_3_)_2_). (**b**) Enlarged region of the Amide I (C=O stretching vibration) at 1630 cm^−1^ and Amide II (ν C-N + δ N-H) vibrations at 1568 cm^−1^. The band positions of the C=O stretching vibrations indicate that all urea groups within the elastomers were hydrogen-bonded in an ordered manner [45,67,68].

**Figure A5:**
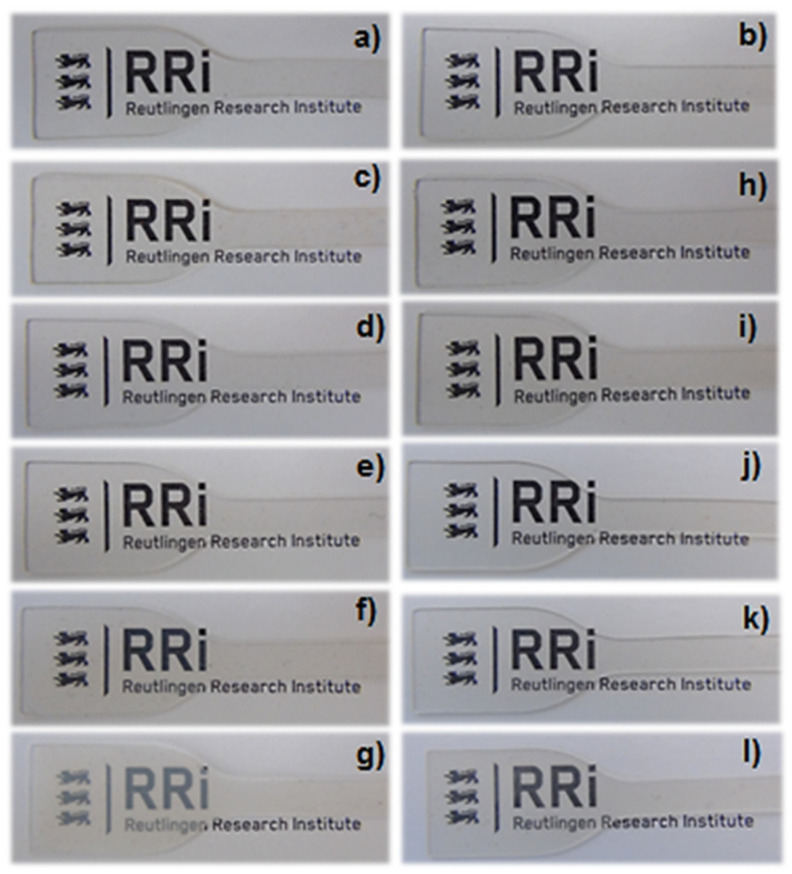
Photographs of PDMS-based PUr elastomer samples used for tensile testing. (**a**) PSU MDI-1.5%. (**b**) PSU CHDI-1.0%. (**c**–**g**) PSU H_12_MDI series with HS contents: (**c**) 1.6%, (**d**) 5%, (**e**) 10%, (**f**) 15%, and (**g**) 20%. (**h**–**l**) PSU IPDI series with HS contents: (**h**) 1.4%, (**i**) 5%, (**j**) 10%, (**k**) 15%, and (**l**) 20%. All PSU elastomer films were transparent until a HS content of 10%. The PSU elastomer films became translucent at HS contents of 15% and 20%.

**Figure A7:**
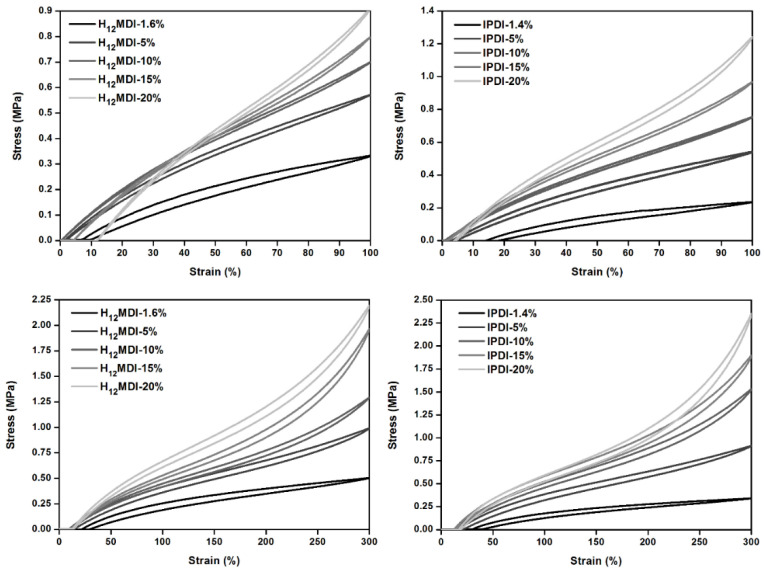
(**top**) 10th cycle hysteresis at 100% strain of PDMS-based polyurea-elastomers synthesized from H_12_MDI and IPDI comprising different HS contents from 1.6% (1.4%) to 20%. (**bottom**) 10th cycle hysteresis at 300% strain.

CE: chain extender (APTMDS); PDI: polydispersity index. ^1^ Intrinsic viscosity was calculated from Huggins plots (see Figure A2 in Appendix B). Values were taken from the intercepts of the fitting curves. ^#^ No linear relationship was obtained for the PSUs H_12_MDI-10% and H_12_MDI-20%. ^2^ The theoretical number average molecular weight of the HSs (g·mol^−1^) was calculated from the reaction stoichiometry. * No chain extender was included in the polymer, and the HS molecular weight resulted from the diisocyanate + terminal urea groups formed by the amino groups of the PDMS. PSU elastomers from H_12_MDI and IPDI were synthesized from a PDMS of ${\bar{M}}_{n}$: 16,338 g·mol^−1^. The PSU copolymers from MDI and CHDI were prepared from a PDMS of ${\bar{M}}_{n}$: 16,296 g·mol^−1^ and ${\bar{M}}_{n}$: 16,472 g·mol^−1^, respectively. (Average values were determined from four titrations).

**Table 3:** Influence of HS content on tensile hysteresis for PSU elastomers synthesized from H_12_MDI with HSs ranging from 1.6% (copolymer) to 20%. The values are given for the first three cycles and the last (10th) cycle at 100% and 300% strain, respectively. The given values are the mean values from three repeated measurements and were rounded to integers.

PSU (Elongation)/Cycle	1	2	3	10
H_12_MDI-1.6% (100%)	24	16	16	15
H_12_MDI-1.6% (300%)	24	17	16	15
H_12_MDI-5% (100%)	11	7	6	5
H_12_MDI-5% (300%)	21	12	11	9
H_12_MDI-10% (100%)	9	4	3	3
H_12_MDI-10% (300%)	21	10	8	7
H_12_MDI-15% (100%)	12	4	3	3
H_12_MDI-15% (300%)	29	11	9	7
H_12_MDI-20% (100%)	28	5	5	4
H_12_MDI-20% (300%)	28	10	9	7

**Table 4:** Influence of HS content on tensile hysteresis for PSU elastomers synthesized from IPDI with HS contents ranging from 1.4% (copolymer) to 20%. The values are given for the first three cycles and the last (10th) cycle at 100% and 300% strain. The given values are the mean values from three repeated measurements and are rounded to integers.

PSU (Elongation)/Cycle	1	2	3	10
IPDI-1.4% (100%)	34	21	20	20
IPDI-1.4% (300%)	26	18	17	16
IPDI-5% (100%)	17	10	9	8
IPDI-5% (300%)	27	15	13	11
IPDI-10% (100%)	10	5	4	3
IPDI-10% (300%)	25	10	9	7
IPDI-15% (100%)	13	5	4	3
IPDI-15% (300%)	31	11	10	8
IPDI-20% (100%)	22	8	7	5
IPDI-20% (300%)	42	13	12	9

**Table 5:** Influence of HS content on tensile hysteresis of PSU copolymers synthesized from the diisocyanates H_12_MDI, IPDI, MDI, and CHDI. The values are given for the first three cycles and the last (10th) cycle at 100% and 300% strain. The given values are the mean values from three repeated measurements and are rounded to integers.

PSU (Elongation)/Cycle	1	2	3	10
H_12_MDI-1.6% (100%)	24	16	16	15
H_12_MDI-1.6% (300%)	24	17	16	15
IPDI-1.4% (100%)	34	21	20	20
IPDI-1.4% (300%)	26	18	17	16
MDI-1.5% (100%)	23	16	15	14
MDI-1.5% (300%)	32	22	20	18
CHDI-1.0% (100%)	19	12	11	9
CHDI-1.0% (300%)	37	21	18	13

**Figure A3:**
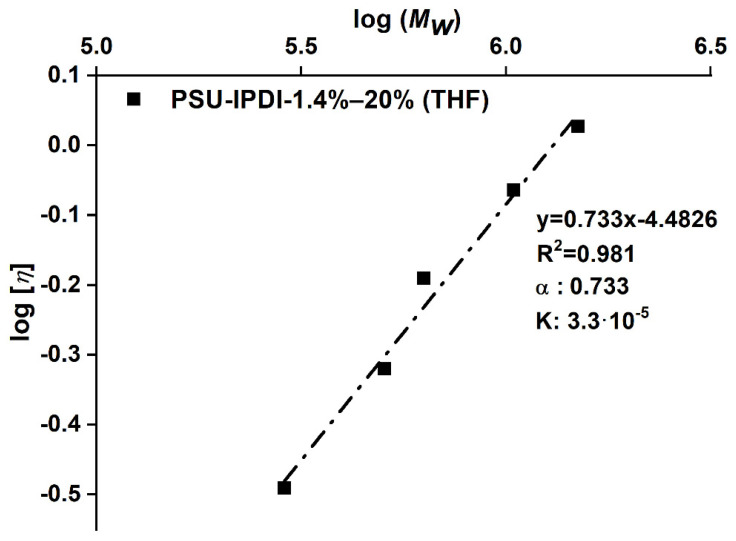
Double logarithmic plot of intrinsic viscosity [*η*] versus weight average molecular weight ${\bar{M}}_{w}$ of the PSU IPDI elastomers determined from SEC. The Mark–Houwink parameters *α* and *K* were obtained as slope and intercept from the linear fitting curve, and were 0.733 and 3.3 × 10^−5^, respectively.

**Figure A6:**
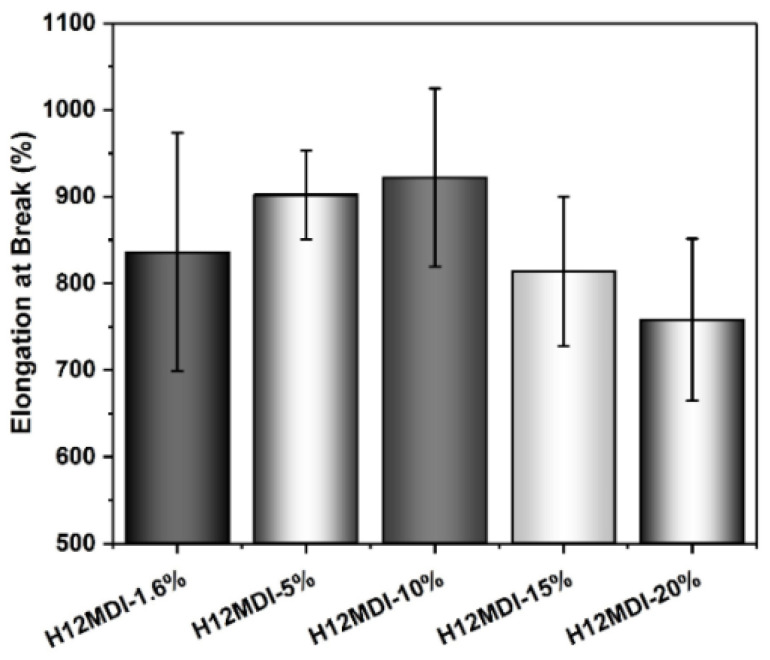

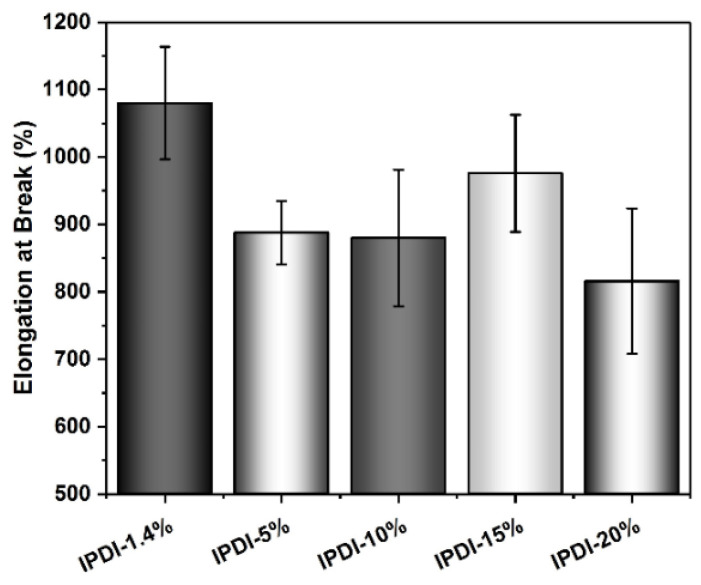
Elongation at break of PDMS-based polyurea-elastomers synthesized from H_12_MDI (**top**) and IPDI (**bottom**) comprising HS contents between 1.6% (1.4%) and 20%.

## Data Availability

Data are available at Reutlingen University.

## Appendix B. Determination of Intrinsic Viscosity of the Polydimethylsiloxane-Based PUr Elastomers

Intrinsic viscosity of the PDMS-based polyurea-elastomers was determined using an Ubbelohde viscometer. PSU solutions comprising four concentrations (0.5–2.0 g·dL^−1^) were prepared in THF and flow times for each polymer solution were measured (10 replicates) relative to the solvent to calculate the specific viscosity (*η_spec._*) according to Equation (A1).

(A1) $$\eta_{spec.}=\frac{\eta_{PS}-\eta_{solvent}}{\eta_{solvent}}\equiv \eta_{spec.}=\frac{t_{PS}-t_{solvent}}{t_{solvent}}$$

were *t_PS_* and *t_solvent_* are the measured times (s) of a defined volume of the polymer solution/solvent passing the capillary.

The reduced viscosity *η_red._* is obtained, when dividing *η_spec._* by the concentration of the polymer solution.

(A2) $$\eta_{red.}=\frac{\eta_{spec.}}{c}$$

The intrinsic viscosity [*η*] is a measure of the capability of a polymer in solution to increase the viscosity of the solution and is obtained when extrapolating *η_spec._* to a concentration of zero.

(A3) $$\left[\eta \right]=\underset{n\to 0}{\lim}\frac{\eta_{spec.}}{c}$$

According to the relation of reduced viscosity and intrinsic viscosity, which was described by Huggins, [*η*] can be calculated as intercept of a fitting curve where reduced viscosity is plotted against the polymer concentration within the solution. *k*_2_ is called Huggins viscosity constant and is a measure of the solvent quality, i.e., if the selected solvent is a good or poor solvent for the polymer [66].

(A4) $$\eta_{red.}=\frac{\eta_{spec.}}{c}=\left[\eta \right]+k_{2}{\left[\eta \right]}^{2}c$$

The Mark–Houwink–Sakurada equation relates the intrinsic viscosity [*η*] to the viscosity-average molecular weight ${\bar{M}}_{v}$ of a polymer. If the parameters *K* and *α* (for a specific polymer-solvent system) are known, one can approximately calculate the weight-average molecular weight ${\bar{M}}_{w}$ of the polymer.

(A5) $$\left[\eta \right]=K\cdot M_{v}^{\alpha }$$

In case of polymer-solvent systems, where the Mark–Houwink parameters are not reported, the parameters can be calculated by plotting the logarithm of intrinsic viscosity against the logarithm of molecular weight. The parameters *K* and *α* are derived as intercept and slope of the linear curve.

(A6) log[η]=logK+α·logM

## Appendix E. Mechanical Properties of Polydimethylsiloxane-Based PUr Elastomers Comprising Varying HS Contents and Different Diisocyanates

**Table A1:** Young’s modulus, ultimate tensile strength, and elongation at break of PDMS-based polyurea elastomers synthesized from the aliphatic diisocyanates H_12_MDI, IPDI, and CHDI, and from the aromatic diisocyanate MDI. Values are given as an average of five repeated measurements ± standard deviation.

PSU	Young’s Modulus (MPa)	Ultimate Tensile Strength (MPa)	Elongation at Break (%)
PSU H_12_MDI-1.6%	0.52 ± 0.05	1.34 ± 0.13	836 ± 137
PSU H_12_MDI-5%	0.83 ± 0.07	2.41 ± 0.14	902 ± 51
PSU H_12_MDI-10%	0.96 ± 0.13	4.07 ± 0.16	922 ± 103
PSU H_12_MDI-15%	1.17 ± 0.07	5.37 ± 0.40	814 ± 86
PSU H_12_MDI-20%	1.34 ± 0.06	6.70 ± 0.52	758 ± 93
PSU IPDI-1.4%	0.27 ± 0.01	1.00 ± 0.07	1080 ± 84
PSU IPDI-5%	0.60 ± 0.04	1.88 ± 0.13	888 ± 47
PSU IPDI-10%	0.92 ± 0.04	3.04 ± 0.19	880 ± 101
PSU IPDI-15%	1.12 ± 0.03	4.56 ± 0.38	976 ± 87
PSU IPDI-20%	1.28 ± 0.10	4.76 ± 0.50	816 ± 108
PSU MDI-1.5%	0.48 ± 0.04	1.36 ± 0.25	1050 ± 140
PSU CHDI-1.0%	0.91 ± 0.05	1.19 ± 0.12	602 ± 77

**Table A2:** Tensile hysteresis at 100% strain of PDMS-based polyurea elastomers synthesized from 4,4-methylenebis(cyclohexyl isocyanate) (H_12_MDI) with HS contents between 1.6% and 20%. Values are given as an average of three repeated measurements. SD: standard deviation.

PSU/Cycle	1	2	3	4	5	6	7	8	9	10
H_12_MDI-1.6%	23.8	16.4	15.5	15.1	14.7	14.7	14.6	14.5	14.4	14.5
SD	1.8	0.2	0.2	0.3	0.2	0.2	0.2	0.2	0.2	0.2
H_12_MDI-5%	11.2	6.9	6.1	5.7	5.5	5.3	5.3	5.2	5.0	5.0
SD	0.8	0.3	0.3	0.4	0.4	0.4	0.4	0.4	0.3	0.4
H_12_MDI-10%	8.8	3.7	3.3	3.0	2.9	2.8	2.7	2.7	2.7	2.6
SD	1.7	0.4	0.3	0.3	0.3	0.3	0.2	0.3	0.3	0.3
H_12_MDI-15%	12.3	3.6	3.2	3.0	2.8	2.8	2.7	2.7	2.6	2.6
SD	4.7	0.2	0.1	0.1	0.1	0.1	0.1	0.1	0.1	0.1
H_12_MDI-20%	27.8	5.1	4.5	4.3	4.2	4.1	4.1	4.0	4.0	4.0
SD	3.5	1.3	1.4	1.5	1.5	1.5	1.5	1.6	1.5	1.5

**Table A3:** Tensile hysteresis at 100% strain of PDMS-based polyurea elastomers synthesized from isophorone diisocyanate (IPDI) with HS contents between 1.4% and 20%. Values are given as an average of three repeated measurements. SD: standard deviation.

PSU/Cycle	1	2	3	4	5	6	7	8	9	10
IPDI-1.4%	34.2	20.9	20.3	20.1	20.0	19.9	19.9	20.0	20.1	20.1
SD	1.8	0.7	0.7	0.6	0.5	0.4	0.5	0.5	0.6	0.4
IPDI-5%	17.0	10.3	9.4	9.1	8.8	8.7	8.5	8.5	8.4	8.3
SD	0.3	0.6	0.7	0.7	0.8	0.8	0.8	0.8	0.8	0.8
IPDI-10%	9.5	4.6	4.0	3.7	3.4	3.3	3.3	3.2	3.1	3.1
SD	0.6	0.3	0.3	0.3	0.3	0.2	0.2	0.2	0.2	0.2
IPDI-15%	13.2	5.1	4.4	4.0	3.9	3.7	3.7	3.6	3.5	3.4
SD	0.4	0.1	0.1	0.1	0.0	0.1	0.1	0.1	0.1	0.1
IPDI-20%	21.6	7.9	6.8	6.3	6.0	5.8	5.6	5.5	5.4	5.3
SD	0.6	0.3	0.2	0.2	0.2	0.2	0.2	0.2	0.2	0.2

**Table A4:** Tensile hysteresis at 100% strain of PDMS-based polyurea copolymers synthesized from 4,4′-methylenebis(phenyl isocyanate) (MDI) and *trans*-1,4-cyclohexane diisocyanate (CHDI) with urea HSs of approximately 1.5% and 1.0%. Values are given as an average of three repeated measurements. SD: standard deviation.

PSU/Cycle	1	2	3	4	5	6	7	8	9	10
MDI-1.5%	23.4	15.7	15.1	14.6	14.3	14.2	14.1	13.9	13.8	13.7
SD	0.8	0.7	0.7	0.6	0.8	0.9	0.9	0.7	0.8	0.7
CHDI-1.0%	18.7	12.3	11.1	10.5	10.1	9.9	9.7	9.5	9.3	9.2
SD	1.6	0.7	0.7	0.8	0.9	0.8	0.8	0.8	0.9	0.9

**Table A5:** Tensile hysteresis at 300% strain of PDMS-based polyurea elastomers synthesized from 4,4-methylenebis(cyclohexyl isocyanate) (H_12_MDI) with HS contents between 1.6% and 20%. Values are given as an average of three repeated measurements. SD: standard deviation.

PSU/Cycle	1	2	3	4	5	6	7	8	9	10
H_12_MDI-1.6%	23.9	17.0	15.8	15.4	15.2	15.0	14.9	14.8	14.8	14.8
SD	0.6	0.3	0.2	0.2	0.2	0.2	0.2	0.2	0.2	0.2
H_12_MDI-5%	21.4	12.1	10.8	10.2	9.9	9.6	9.4	9.2	9.1	8.9
SD	1.1	0.2	0.3	0.3	0.3	0.3	0.3	0.3	0.3	0.3
H_12_MDI-10%	21.1	9.5	8.4	7.9	7.5	7.3	7.1	6.9	6.8	6.7
SD	1.3	1.3	1.2	1.2	1.2	1.1	1.1	1.1	1.0	1.0
H_12_MDI-15%	28.9	10.6	9.2	8.6	8.1	7.9	7.6	7.4	7.3	7.1
SD	1.8	0.9	0.8	0.8	0.8	0.7	0.7	0.7	0.6	0.7
H_12_MDI-20%	27.5	10.0	8.7	8.2	7.8	7.6	7.3	7.2	7.1	7.0
SD	0.9	0.3	0.3	0.5	0.4	0.4	0.3	0.3	0.4	0.5

**Table A6:** Tensile hysteresis at 300% strain of PDMS-based polyurea elastomers synthesized from isophorone diisocyanate (IPDI) with HS contents between 1.4% and 20%. Values are given as an average of three repeated measurements. SD: standard deviation.

PSU/Cycle	1	2	3	4	5	6	7	8	9	10
IPDI-1.4%	25.6	18.1	17.2	16.8	16.6	16.5	16.4	16.4	16.3	16.4
SD	0.9	0.3	0.5	0.5	0.5	0.6	0.6	0.6	0.7	0.7
IPDI-5%	26.9	14.5	13.1	12.3	11.9	11.6	11.4	11.2	11.1	10.9
SD	2.1	0.8	0.9	0.9	0.9	1.0	1.0	1.0	1.0	1.0
IPDI-10%	25.0	10.0	8.7	8.1	7.8	7.5	7.3	7.2	7.0	6.9
SD	0.9	0.2	0.2	0.3	0.3	0.3	0.4	0.4	0.4	0.4
IPDI-15%	30.7	11.2	9.6	9.0	8.6	8.3	8.1	7.9	7.7	7.6
SD	1.7	0.1	0.1	0.1	0.1	0.1	0.1	0.1	0.1	0.2
IPDI-20%	41.5	13.4	11.5	10.6	10.0	9.7	9.4	9.2	9.0	8.8
SD	0.9	0.2	0.1	0.1	0.1	0.1	0.1	0.1	0.1	0.2

**Table A7:** Tensile hysteresis at 300% strain of PDMS-based polyurea copolymers synthesized from 4,4′-methylenebis(phenyl isocyanate) (MDI) and *trans*-1,4-cyclohexane diisocyanate (CHDI) with urea HSs of approximately 1.5% and 1.0%. Values are given as an average of three repeated measurements. SD: standard deviation.

PSU/Cycle	1	2	3	4	5	6	7	8	9	10
MDI-1.5%	32.0	22.1	19.9	19.1	18.8	18.5	18.3	18.1	17.9	17.8
SD	1.5	1.1	0.6	0.7	0.7	0.7	0.7	0.6	0.7	0.8
CHDI-1.0%	37.4	20.6	17.9	16.6	15.9	15.3	14.7	14.3	13.8	13.1
SD	0.9	0.5	0.5	0.4	0.3	0.3	0.3	0.3	0.3	0.3

## Appendix F. Film Thicknesses of Polydimethylsiloxane-Based PUr Elastomers Used for UV–Vis Spectroscopy

**Table A8:** Film thicknesses of PSU films measured using a Mitutoyo Digimatic Thickness Gauge Series 547. Values are given as an average from three measurements.

PSU	Film Thickness (mm)
H_12_MDI-1.6%	0.36
H_12_MDI-5%	0.33
H_12_MDI-10%	0.36
H_12_MDI-15%	0.34
H_12_MDI-20%	0.35
IPDI-1.4%	0.34
IPDI-5%	0.35
IPDI-10%	0.35–0.36
IPDI-15%	0.35–0.36
IPDI-20%	0.36
MDI-1.5%	0.35
CHDI-1.0%	0.36
